# Association between the C-reactive protein-albumin-lymphocyte index and cardiovascular incidence and mortality among patients with chronic kidney disease: a prospective study

**DOI:** 10.3389/fimmu.2026.1729647

**Published:** 2026-02-06

**Authors:** Chen Jiang, Qi Zhou, Jian Feng, Shuo Liu, Miaomiao Fan, Hao Bai, Shujuan Lin, Liyong Chen

**Affiliations:** 1 Department of Nutrition, Qilu Hospital of Shandong University, Jinan, China; 2 Department of Health, Shandong University of Traditional Chinese Medicine, Jinan, China; 3 Department of Gastroenterology, Qilu Hospital of Shandong University, Jinan, China; 4 Department of Geriatric Medicine, Qilu Hospital of Shandong University, Jinan, Shandong, China; 5 Clinical Epidemiology Unit, Qilu Hospital of Shandong University, Jinan, Shandong, China; 6 School of Basic Medicine Science, Key Laboratory of Translational Tumor Medicine in Fujian Province, Putian University, Putian, China; 7 Department of Toxicology and Nutrition, School of Public Health, Cheeloo College of Medicine, Shandong University, Jinan, China

**Keywords:** all-cause mortality, cardiovascular disease, C-reactive protein-to-albumin-to-lymphocyte index, nonlinear, UK Biobank

## Abstract

**Background and aim:**

The recently developed C-reactive protein-to-albumin-to-lymphocyte (CALLY) index represents a novel composite biomarker that simultaneously reflects inflammatory status, immune competence, and nutritional adequacy. However, to date, there are limited evidence on whether CALLY index affects the cardiovascular disease (CVD) events in patients with chronic kidney disease (CKD). This prospective cohort study aimed to investigate the associations between the CALLY index and CVD incidence, all-cause and CVD-specific mortality in CKD patients from UK Biobank.

**Methods:**

The CALLY index was calculated based on lymphocyte counts, serum albumin concentrations, and C-reactive protein (CRP) levels. The association between CALLY index and diverse CVD events were analyzed using multivariate Cox proportional hazards regression and restricted cubic splines (RCS) analysis.

**Results:**

A total of 22,898 CKD patients were included. Compared to participants with lowest quartile of CALLY, those with highest quartile had decreased risk of incident overall CVD (HR: 0.70, 95%CI: 0.65-0.75), IHD (HR: 0.70, 95%CI: 0.63-0.78), MI (HR: 0.69, 95%CI: 0.57-0.83), stroke (HR: 0.74, 95%CI: 0.62-0.88), and all-cause (HR: 0.55, 95%CI: 0.51-0.61) and CVD-specific mortality (HR: 0.54, 95%CI: 0.44-0.66). RCS analysis showed the significant L-shaped dose-response relationships between CALLY index and CVD incidence and mortality outcomes, indicating a saturation effect.

**Conclusion:**

The moderate-to-high CALLY index was significantly associated with a reduced risk of CVD events-including IHD, MI, and stroke, as well as lower all-cause and CVD-specific mortality. These findings suggest that the CALLY index, which integrates inflammatory, nutritional, and immunological markers, may serve as a potential biomarker for risk stratification in patients with CKD. Future longitudinal studies incorporating repeated assessments would be valuable to better characterize the temporal trajectory of the CALLY index and its association with cardiovascular events and mortality in patients with CKD, thereby improving causal inference.

## 1 Introduction

Chronic kidney disease (CKD), characterized by persistent structural or functional renal impairment with reduced glomerular filtration rate (GFR) (1), has emerged as a global health crisis affecting approximately 850 million individuals worldwide (2). CKD currently ranks among the fastest-growing causes of mortality globally, with projections indicating it will become the fifth leading contributor to years of life lost by 2040 (3). Of particular clinical significance, CKD has been firmly established as an independent risk factor for cardiovascular disease (CVD) - the predominant driver of both morbidity and mortality in this vulnerable patient population (4).

The pathophysiology of CVD in CKD patients involves a complex interplay of inflammation, nutritional deficiencies, and immune dysregulation (5, 6). Emerging evidence suggests that anti-inflammatory and immunomodulatory strategies may offer cardiovascular benefits (7), while dietary modifications and lifestyle interventions have demonstrated efficacy in CVD prevention (8). Consequently, integrated evaluation of nutritional, inflammatory, and immunological parameters may provide valuable insights for reducing CVD risk in CKD patients. Clinical studies have identified serum albumin as an independent predictor of all-cause and cardiovascular mortality (9), while C-reactive protein (CRP) has been widely validated as a robust biomarker for cardiovascular risk assessment. Furthermore, growing evidence implicates immune mechanisms in CVD pathogenesis, with lymphocyte counts emerging as significant prognostic indicators (10). Therefore, an indicator capable of integrating and reflecting these interconnected pathological processes may provide a more precise assessment of cardiovascular risk in CKD patients than any single parameter. The recently developed C-reactive protein-to-albumin-to-lymphocyte (CALLY) index represents a novel composite biomarker that simultaneously reflects inflammatory status (CRP), immune competence (lymphocytes), and nutritional adequacy (albumin) (11). Its design is grounded in robust pathophysiological rationale. CRP is a key acute-phase protein and a classic marker of systemic inflammation. As a central mediator of systemic inflammation, CRP directly drives endothelial dysfunction and atherosclerosis (12). Elevated serum CRP levels are associated with an increased risk of CKD (13). Serum albumin is not only a marker of nutritional reserves but also a negative acute-phase reactant; its synthesis decreases while its catabolism increases under inflammatory conditions, and decreased levels are closely associated with protein-energy wasting and poor prognosis (14, 15). Lymphocyte count reflects the state of adaptive immunity; in the context of CKD-associated chronic inflammation and uremia, it is prone to exhaustion and is closely linked to poor prognosis (16). The integration of these three components into the CALLY index theoretically provides a more comprehensive reflection of CVD risk in CKD patients. In recent years, emerging empirical evidence also supports the application value of the CALLY index in this context.

Recent studies shows that a higher CALLY index is an independent protective factor associated with reduced all-cause mortality in patients undergoing maintenance hemodialysis (17). Studies in patients with cardiorenal syndrome (CRS) also indicate that the CALLY index is inversely associated with CRS risk, and its predictive capability is superior to that of traditional inflammatory markers (18). Furthermore, derived from routinely available clinical parameters, this index offers practical advantages of accessibility and ease of calculation (19). Retrospective studies have demonstrated the clinical utility of the CALLY index across various conditions, showing inverse associations with peripheral artery disease (PAD) (20), depression (21), and stroke risk in hypertensive patients (22). Moreover, prior studies have shown superior prognostic performance compared to conventional biomarkers such as systemic immune-inflammation index (SII), neutrophil-to-lymphocyte ratio (NLR), platelet-to-lymphocyte ratio (PLR), and lymphocyte-to-monocyte ratio (LMR) in CRS (18), hepatocellular carcinoma (19), colorectal cancer (23), and post - percutaneous coronary intervention (PCI) coronary artery disease (CAD) (24), suggesting its potential as a more comprehensive risk assessment tool. However, to date, there are limited evidence on whether CALLY index affects the incidence of CVD and mortality among patients with CKD.

To address this knowledge gap, we utilized data from the UK Biobank to investigate the associations between the CALLY index and CVD incidence, all-cause and CVD-specific mortality in patients with CKD. In addition, we conducted comparative analyses against established inflammatory markers, including the SII, NLR, PLR, and LMR, aiming to establish its relative prediction superiority.

## 2 Methods

### 2.1 Study population

The UK Biobank is a large-scale, population-based prospective cohort study that recruited over 500,000 participants aged 40–69 years across the United Kingdom between 2006 and 2010. With a median follow-up period of 12 years, this comprehensive investigation aims to identify key determinants of disease development and progression. The study integrates multidimensional data, including detailed sociodemographic characteristics, lifestyle factors (such as dietary habits, physical activity levels, and smoking status), family medical history, cognitive assessments, comprehensive physiological measurements, biological samples, and advanced imaging data. Health outcomes are systematically tracked through robust linkages to national electronic health records, encompassing mortality data, cancer registry information, hospital admission records, and primary care documentation, which enables the study to capture a wide spectrum of major diseases, including cardiovascular disorders, diabetes, and various malignancies (25, 26). This study was conducted using the UK Biobank Resource (Application ID: 95817).

In this study, we identified 34,925 participants with CKD. Individuals who met any of the following criteria defined as having CKD (1): estimated glomerular filtration rate (eGFR) < 60 mL/min/1.73m^2^ (2); albuminuria (albumin to creatinine ratio [ACR] ≥ 30 mg/g); 3) CKD diagnosis based on International Classification of Diseases-10 (ICD-10, codes N18) (27). Individuals with missing lymphocyte counts (n = 1,582), missing albumin measurements (n = 3,991), missing CRP values (n = 129), and those with baseline CVD (n = 6,325) were excluded. Ultimately, 22,898 eligible participants were included for analyses of CALLY index and CVD incidence (including IHD, MI, and stroke), all-cause mortality, and CVD-specific mortality among individuals with CKD.

### 2.2 Assessment of CALLY index

The CALLY index is a composite metric integrating serum albumin levels (g/L), lymphocyte counts (10^^9^/L), and CRP values (mg/L) to provide a comprehensive assessment of systemic inflammatory and nutritional status, with detailed biomarker measurement descriptions available in the UK Biobank online showcase (https://biobank.ndph.ox.ac.uk/showcase). Blood samples collected at baseline (2006–2010) were analyzed using standardized methods: serum albumin was measured via the BCG method on a Beckman Coulter AU5800 analyzer; lymphocyte count was derived from the “Lymphocyte Number” assay of UK Biobank, calculated as (lymphocytes/100) × white blood cell count; and CRP was quantified using a high-sensitivity immunoturbidimetric assay on a Beckman Coulter AU5800 platform. The index was calculated according to the established formula (19):


CALLY index = Albumin (g/L) × Lymphocyte (10^9/L)]/CRP (mg/L) × 10

### 2.3 Ascertainment of outcomes

The primary outcomes were the incidence of overall CVD, including IHD, stroke, and MI among patients with CKD. The secondary outcome was all-cause and CVD-specific mortality. Participants were followed from the time of enrollment to the date of incident CVD, loss to follow-up, death, or July 8, 2024, which ever occurred first. In this study, CVD were identified using the International Classification of Diseases, 10th Revision (ICD-10) codes: including I20-I64 for overall CVD events, I20-I25 for IHD, I21-I23 for MI, I60-I64 for stroke, and I00-I99 for death resulting from CVD (27).

### 2.4 Assessment of covariates

At baseline enrollment, we systematically collected data on a comprehensive set of covariates, which we categorized into demographic characteristics, socioeconomic factors, lifestyle factors, anthropometric measurements, and medical condition. Demographic variables included age, sex (male/female), and self-reported ethnicity (categorized as White, Mixed, South Asian, Black, Chinese, or other). Socioeconomic status was assessed using the highest attained educational qualification (grouped as no relevant qualifications, college or university degree, A levels/AS levels or equivalent, O levels/GCSEs or equivalent, CSEs or equivalent, or others) and the Townsend deprivation index, an area-based measure of socioeconomic deprivation. For lifestyle factors, smoking status was classified as current, former, or never smoker and alcohol drinking status was categorized as never, past, or current drinking. Healthy diet score was assessing using a definition of ideal intake of healthy and unhealthy dietary components for cardiovascular, which includes increased consumption of fruits, vegetables, whole grains, fish, dairy products and vegetable oils, and reduced or no consumption of refined grains, processed or unprocessed meats and sugar-sweetened beverages (28). To assess leisure-time physical activity, participants were asked: “In the past four weeks, have you engaged in any of the following activities: walking for pleasure, light do-it-yourself (DIY) projects, heavy DIY work, strenuous sports, other exercises, or none of the above?” For those who reported physical activity, information on both frequency and duration was collected. We quantified the intensity of these leisure-time activities using metabolic equivalent of task (MET) values, which represent the ratio of energy expenditure (per kilogram of body weight per hour) to the standard resting metabolic rate. The total weekly leisure-time physical activity was calculated in MET-minutes per week by multiplying the frequency, duration, and corresponding MET values for each activity. The assigned MET values were as follows: 3.5 for walking for pleasure, 2.5 for light DIY, 5.5 for heavy DIY, 8.0 for strenuous sports, and 4.0 for other physical activities (29, 30). Body mass index (BMI) was calculated as weight (kg) divided by height squared (m²). Renal function was assessed using eGFR (mL/min/1.73 m²) Medical conduction included self-reported or physician-diagnosed conditions such as diabetes, hypertension, and hypercholesterolemia. Family history of CVD was recorded as a binary variable (yes or no) based on reports of CVD in first-degree relatives.

### 2.5 Statistical analysis

The study population was categorized into quartiles (Q1-Q4) based on CALLY index distribution. Baseline characteristics were reported as mean (standard deviation [SD]) for continuous variables and number (percentage) for categorical variables. Baseline characteristics across CALLY index quartiles were compared using one-way ANOVA for continuous variables and Chi-square test for categorical variables. Participants with missing data for any key component of the CALLY index were excluded from the analysis. For the remaining covariates, missing data were handled as follows: categorical variables (including categorized continuous variables) assigned missing values to a separate “missing” category, which was then included in the regression models. This strategy was employed to preserve the overall sample size and statistical power of the analysis.

Multivariable Cox proportional hazards models were used to assess the association of the CALLY index with risk of CVD incidence and mortality among CKD patients. Hazard ratios (HRs) with 95% confidence intervals (CIs) for CVD incidence (including IHD, MI, and stroke), were evaluated through three sequential adjusted models: crude model was unadjusted, model 1 was adjusted for age, sex, ethnicity (White, mixed background, south Asian, Black, Chinese, or other), educational attainment (no relevant qualifications, college or University degree, A levels/AS levels or equivalent, O levels/GCSEs or equivalent, CSEs or equivalent, or others), and socioeconomic deprivation; model 2 was further adjusted for BMI, smoking status (never, previous, or current), alcohol drinking (never, previous, or current), healthy diet, leisure time physical activity (<500, 500 to <1000, or ≥1000 MET mins/week), eGFR, family history of CVD, diabetes, hypertension, and hypercholesterolemia. The proportional hazards assumption was assessed using Schoenfeld residuals and was found to hold for all final models. The confounders included in the models were selected based on prior published evidence (27, 31), whereby covariates associated with the outcome or those that altered the effect estimate of the primary exposure by more than 10% were retained (32). To evaluate the presence of multicollinearity, we calculated the Variance Inflation Factor (VIF) for all variables included in the regression models. Kaplan-Meier curves were generated to compare cumulative incidence of CVD and mortality across quartiles of the CALLY index. Differences between survival curves were evaluated using the log-rank test. Furthermore, dose-response relationships were analyzed using restricted cubic splines (RCS) to assess potential nonlinear associations between the CALLY index and CVD incidence and mortality among CKD patients. We further applied two-segmented Cox proportional hazard models to examine the threshold effect of CALLY index on CVD incidence and mortality. Log-likelihood ratio test comparing one-line (non-segmented) model to segmented regression model was used to determine whether threshold exists. Subgroup analyses according to the covariates and sensitivity analyses excluding participants with less than 2 years of follow-up were conducted to evaluate the robustness of observed associations. To test the independent predictive capacity of the CALLY index, we calculated the Harrell’s C-index using bootstrap methods with 500 replications. The statistical comparison of the Harrell’s C-index among the four biomarkers was performed using the “compareC” package. All statistical analyses were performed using R version 4.2.2 (R Foundation for Statistical Computing). A two-sided p-value <0.05 defined statistical significance.

## 3 Results

### 3.1 Baseline characteristics of the study population

**Table 1** presents the baseline characteristics of participants stratified by CALLY index quartiles. The study comprised a cohort of 22,898 individuals with CKD, with a mean age of 59.21 ± 7.72 years, including 10,061 (43.9%) males. Compared to participants in the lowest quartile of the CALLY index, those in the highest quartile were younger, more likely to be female, of non-White ethnicity, and have a healthy diet. They also exhibited higher educational attainment, greater engagement in leisure-time physical activity, and elevated eGFR levels, and tend to had a lower socioeconomic deprivation index, BMI, and proportion of never-smokers, and had higher proportion of current drinkers. Furthermore, they were less likely to report a family history of CVD, diabetes, hypertension, or hypercholesterolemia. Among 22,898 participants included in the study, a total of 7,063 incident CVD events were observed, comprising 3,217 cases of IHD, 1,004 cases of MI, and 1,128 strokes. By the study exit date, 4247 (18.55%) individuals in the cohort had died; of those deaths, 973 (4.25%) were attributed to CVD.

**Table 1:** Baseline characteristics of individuals with chronic kidney disease according to CALLY index in the UK Biobank.

Variables	Total population (n = 22,898)	Quartiles of CALLY index				
		Q1 (n = 5725)	Q2 (n = 5724)	Q3 (n = 5724)	Q4 (n = 5725)	P-value
Age, mean (SD), years	59.21 ± 7.72	59.64 ± 7.65	59.68 ± 7.47	59.36 ± 7.67	58.14 ± 7.99	<0.001
Male, n (%)	10061 (43.94)	2470 (43.14)	2626 (45.88)	2576 (45.00)	2389 (41.73)	<0.001
Ethnicity, n (%)						<0.001
White	21087 (92.59)	5312 (93.27)	5321 (93.42)	5289 (92.87)	5165 (90.79)	
Mixed background	143 (0.63)	30 (0.53)	37 (0.65)	40 (0.70)	36 (0.63)	
South Asian	680 (2.99)	159 (2.79)	154 (2.70)	160 (2.81)	207 (3.64)	
Black	503 (2.21)	118 (2.07)	107 (1.88)	110 (1.93)	168 (2.95)	
Chinese	79 (0.35)	10 (0.18)	10 (0.18)	18 (0.32)	41 (0.72)	
Other	283 (1.24)	66 (1.16)	67 (1.18)	78 (1.37)	72 (1.27)	
Educational attainment, n (%)						<0.001
No relevant qualifications	5282 (23.47)	1613 (28.74)	1413 (25.10)	1231 (21.83)	1025 (18.21)	
College or University degree	6160 (27.37)	1227 (21.86)	1402 (24.90)	1596 (28.31)	1935 (34.38)	
A levels/AS levels or equivalent	2232 (9.92)	503 (8.96)	560 (9.95)	592 (10.50)	577 (10.25)	
O levels/GCSEs or equivalent	4810 (21.37)	1237 (22.04)	1197 (21.26)	1203 (21.34)	1173 (20.84)	
CSEs or equivalent	1107 (4.92)	273 (4.86)	275 (4.88)	286 (5.07)	273 (4.85)	
Others	2918 (12.96)	760 (13.54)	783 (13.91)	730 (12.95)	645 (11.46)	
Socioeconomic deprivation, mean (SD)	-1.05 ± 3.20	-0.69 ± 3.33	-1.03 ± 3.16	-1.24 ± 3.11	-1.22 ± 3.15	<0.001
Body mass index, mean (SD), kg/m2	28.66 ± 5.52	31.10 ± 6.34	29.67 ± 5.21	28.05 ± 4.59	25.84 ± 4.27	<0.001
Smoking status, n (%)						<0.001
Never	11806 (51.91)	2719 (47.85)	2869 (50.46)	3062 (53.80)	3156 (55.52)	
Previous	8327 (36.61)	2229 (39.23)	2145 (37.72)	2058 (36.16)	1895 (33.34)	
Current	2610 (11.48)	734 (12.92)	672 (11.82)	571 (10.03)	633 (11.14)	
Alcohol drinking, n (%)						<0.001
Never	1409 (6.18)	437 (7.67)	350 (6.14)	315 (5.52)	307 (5.38)	
Previous	977 (4.28)	320 (5.61)	240 (4.21)	219 (3.84)	198 (3.47)	
Current	20429 (89.54)	4943 (86.72)	5114 (89.66)	5174 (90.64)	5198 (91.15)	
Healthy diet, n (%)	3999 (18.15)	922 (16.83)	968 (17.58)	991 (17.91)	1118 (20.26)	<0.001
Leisure time physical activity, n (%)						<0.001
<500 MET mins/week	8541 (42.12)	2316 (49.12)	2216 (43.73)	2063 (39.58)	1946 (36.82)	
500 to <1000 MET mins/week	4472 (22.05)	968 (20.53)	1106 (21.83)	1180 (22.64)	1218 (23.05)	
≥1000 MET mins/week	7266 (35.83)	1431 (30.35)	1745 (34.44)	1969 (37.78)	2121 (40.13)	
eGFR, mean (SD), mL/min/1.73 m2	79.03 ± 21.70	77.17 ± 23.37	78.50 ± 21.27	79.08 ± 20.78	81.38 ± 21.09	<0.001
Family history of CVD, n (%)	9080 (39.65)	2256 (39.41)	2305 (40.27)	2296 (40.11)	2223 (38.83)	0.365
Diabetes, n (%)	2854 (12.55)	805 (14.16)	694 (12.21)	692 (12.16)	663 (11.67)	<0.001
Hypertension, n (%)	10739 (46.99)	2980 (52.15)	2881 (50.40)	2663 (46.60)	2215 (38.79)	<0.001
Hypercholesterolemia, n (%)	4518 (19.73)	1031 (18.01)	1082 (18.90)	1225 (21.40)	1180 (20.61)	<0.001
Albumin, mean (SD), g/L	45.00 ± 2.91	43.83 ± 2.96	44.90 ± 2.75	45.39 ± 2.76	45.86 ± 2.78	<0.001
Lymphocyte, mean (SD), 10^9/L	2.02 ± 1.31	1.80 ± 0.66	1.97 ± 0.67	2.06 ± 0.67	2.24 ± 2.32	<0.001
C-reactive protein, mean (SD), mg/L	3.70 ± 5.92	9.88 ± 9.22	2.87 ± 1.14	1.45 ± 0.57	0.60 ± 0.37	<0.001

### 3.2 Association between CALLY index and CVD incidence in patients with CKD

**Figure 1:**
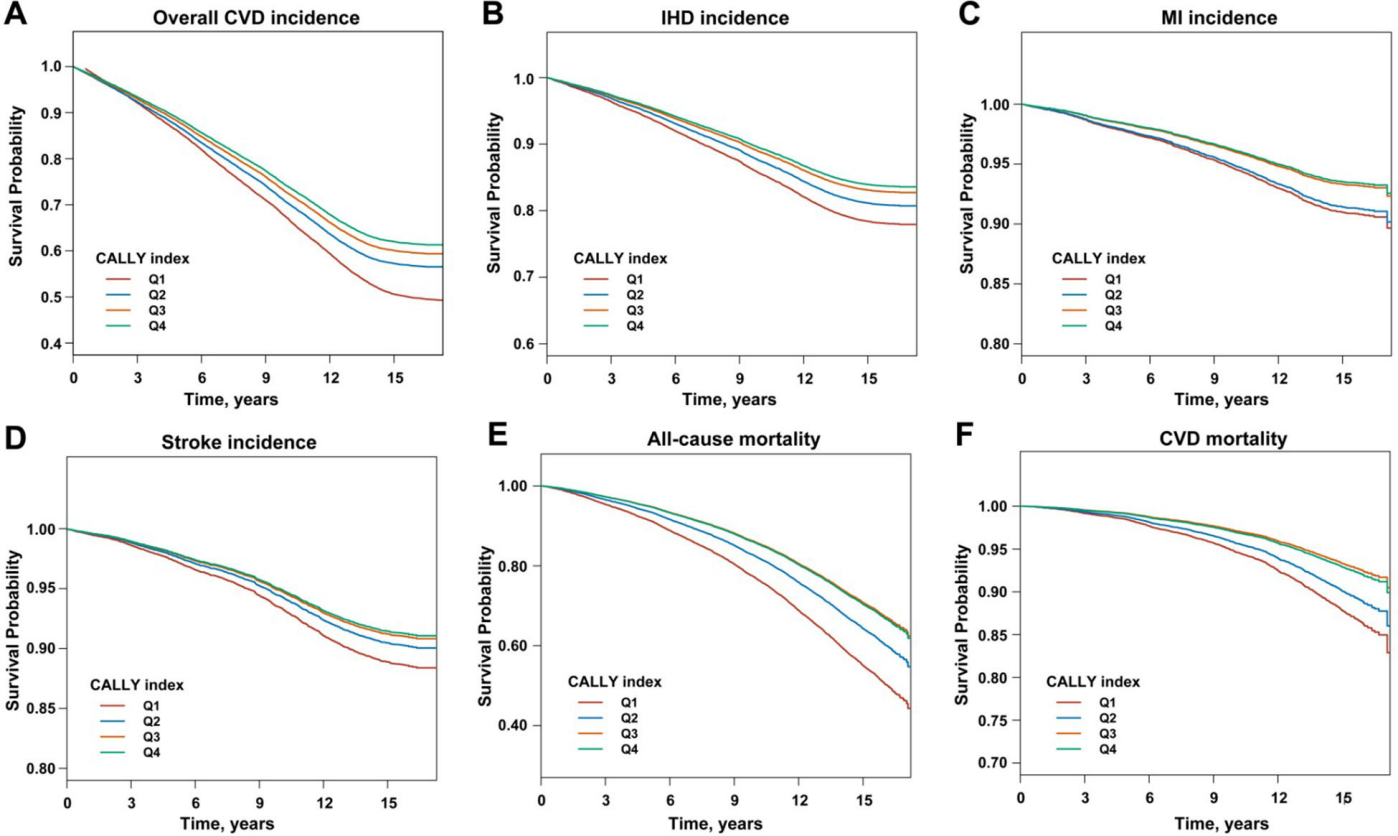
Kaplan-Meier survival curves for CVD incidence and all-cause and CVD-specific mortality stratified by quartiles of CALLY index. **(A)** CVD incidence; **(B)** ischemic heart disease; **(C)** myocardial infarction; **(D)** stroke; **(E)** all-cause mortality; **(F)** CVD-specific mortality.

Associations between CALLY index and CVD incidence are shown in **Table 2**. We constructed three sequential models to assess the associations between the CALLY index and CVD incidence, including overall CVD, IHD, MI, and stroke. The multivariate analysis results showed that per 1-SD increase in CALLY index was associated with a 9% decreased risk of incident overall CVD (HR: 0.91, 95% CI:0.88, 0.94), a 12% decreased risk of incident IHD (HR: 0.88, 95% CI: 0.84,0.93), a 16% reduced risk of incident MI (HR: 0.84, 95% CI: 0.76, 0.93), and a 6% reduced risk of incident stroke (HR: 0.94, 95% CI: 0.87, 1.02). Compared to CKD patients with the lowest quartile of CALLY (Q1), those with the highest quartile of CALLY (Q4) had decreased risk of incident overall CVD (HR: 0.70, 95% CI: 0.65, 0.75), IHD (HR: 0.70, 95% CI: 0.63, 0.78), MI (HR: 0.69, 95% CI: 0.57, 0.83), and stroke (HR: 0.74, 95% CI: 0.62, 0.88). Similar findings were also found in crude model and model 1 (all *P* < 0.001). The VIF values of all exposure and adjustment variables were below 10, indicating that multicollinearity was not a substantial concern in our main model (**Supplementary Table S1**). Kaplan-Meier analysis showed that CKD patients with the lowest quartile of CALLY index, those with higher CALLY had a significantly lower cumulative incidence of overall CVD, IHD, MI, and stroke (log-rank *P* < 0.05) (**Figures 1A-D**).

**Table 2:** Association between CALLY index and CVD incidence in CKD patients.

Outcomes	Quartiles of CALLY index					
	Q1	Q2	Q3	Q4	P for trend	Per 1-SD increase in CALLY index
CVD incidence						
Events	2186	1856 (32.42)	1635 (28.56)	1386 (24.21)		
Crude	1.00 (Ref.)	0.79 (0.74, 0.84)	0.67 (0.63, 0.72)	0.55 (0.52, 0.59)	<0.001	0.80 (0.78, 0.83)
Model 1	1.00 (Ref.)	0.78 (0.73, 0.83)	0.68 (0.64, 0.73)	0.61 (0.57, 0.65)	<0.001	0.86 (0.83, 0.89)
Model 2	1.00 (Ref.)	0.81 (0.77, 0.87)	0.74 (0.70, 0.79)	0.70 (0.65, 0.75)	<0.001	0.91 (0.88, 0.94)
IHD incidence						
Events	1009 (17.62)	870 (15.20)	728 (12.72)	610 (10.66)		
Crude	1.00 (Ref.)	0.82 (0.75, 0.90)	0.67 (0.61, 0.74)	0.55 (0.50, 0.61)	<0.001	0.78 (0.73, 0.82)
Model 1	1.00 (Ref.)	0.81 (0.74, 0.89)	0.68 (0.62, 0.75)	0.61 (0.55, 0.68)	<0.001	0.83 (0.79, 0.88)
Model 2	1.00 (Ref.)	0.85 (0.78, 0.94)	0.75 (0.68, 0.83)	0.70 (0.63, 0.78)	<0.001	0.88 (0.84, 0.93)
MI incidence						
Events	311 (5.43)	295 (5.15)	214 (3.74)	184 (3.21)		
Crude	1.00 (Ref.)	0.91 (0.78, 1.07)	0.64 (0.54, 0.76)	0.55 (0.46, 0.66)	<0.001	0.74 (0.67, 0.81)
Model 1	1.00 (Ref.)	0.90 (0.76, 1.05)	0.66 (0.55, 0.78)	0.61 (0.51, 0.74)	<0.001	0.79 (0.72, 0.88)
Model 2	1.00 (Ref.)	0.95 (0.81, 1.11)	0.72 (0.60, 0.86)	0.69 (0.57, 0.83)	<0.001	0.84 (0.76, 0.93)
Stroke incidence						
Events	331 (5.78)	293 (5.12)	264 (4.61)	240 (4.19)		
Crude	1.00 (Ref.)	0.85 (0.72, 0.99)	0.74 (0.63, 0.87)	0.67 (0.57, 0.80)	<0.001	0.89 (0.82, 0.96)
Model 1	1.00 (Ref.)	0.84 (0.71, 0.98)	0.75 (0.64, 0.89)	0.75 (0.63, 0.88)	<0.001	0.94 (0.88, 1.02)
Model 2	1.00 (Ref.)	0.85 (0.72, 0.99)	0.77 (0.65, 0.91)	0.74 (0.62, 0.88)	<0.001	0.94 (0.87, 1.02)

Model 1 was adjusted for age, sex, ethnicity, educational attainment, and socioeconomic deprivation; Model 2 was further adjusted for body mass index, smoking status, alcohol drinking, healthy diet, leisure time physical activity, eGFR, family history of CVD, diabetes, hypertension, and hypercholesterolemia.

### 3.3 Association between the CALLY index and all-cause and cardiovascular mortality in patients with CKD

As presented in **Table 3**, the multivariate Cox regression models showed that the per 1-SD increase in CALLY index was negatively associated with all-cause mortality (HR: 0.86, 95% CI: 0.82, 0.90) and CVD-specific mortality (HR: 0.83, 95% CI: 0.74, 0.92). Furthermore, compared to CKD patients with the lowest quartile of CALLY (Q1), those with the highest quartile of CALLY (Q4) had a 45% decreased all-cause mortality (HR: 0.55, 95% CI: 0.51, 0.61) and a 46% decreased CVD-specific mortality (HR: 0.54, 95% CI: 0.44, 0.66). These associations remained significant in the crude model and model 1 (all *P* < 0.001). Kaplan-Meier survival curves for all-cause and cardiovascular mortality revealed the significant divergence between high and low CALLY index groups (log-rank P<0.05) (**Figures 1E, F**).

**Table 3:** Association between CALLY index and all-cause and CVD-specific mortality in CKD patients.

Outcomes	Quartiles of CALLY index					
	Q1	Q2	Q3	Q4	P for trend	Per 1-SD increase in CALLY index
All-cause mortality						
Events	1527 (26.67)	1129 (19.72)	842 (14.71)	749 (13.08)		
Crude	1.00 (Ref.)	0.71 (0.65, 0.76)	0.51 (0.47, 0.56)	0.45 (0.42, 0.50)	<0.001	0.77 (0.73, 0.80)
Model 1	1.00 (Ref.)	0.70 (0.65, 0.76)	0.53 (0.49, 0.57)	0.53 (0.49, 0.58)	<0.001	0.84 (0.80, 0.88)
Model 2	1.00 (Ref.)	0.74 (0.68, 0.80)	0.57 (0.52, 0.62)	0.55 (0.51, 0.61)	<0.001	0.86 (0.82, 0.90)
CVD mortality						
Events	355 (6.20)	284 (4.96)	175 (3.06)	159 (2.78)		
Crude	1.00 (Ref.)	0.76 (0.65, 0.89)	0.46 (0.38, 0.55)	0.41 (0.34, 0.50)	<0.001	0.70 (0.63, 0.78)
Model 1	1.00 (Ref.)	0.75 (0.64, 0.88)	0.47 (0.39, 0.56)	0.48 (0.40, 0.58)	<0.001	0.77 (0.69, 0.85)
Model 2	1.00 (Ref.)	0.79 (0.68, 0.93)	0.52 (0.43, 0.62)	0.54 (0.44, 0.66)	<0.001	0.83 (0.74, 0.92)

Model 1 was adjusted for age, sex, ethnicity, educational attainment, and socioeconomic deprivation; Model 2 was further adjusted for body mass index, smoking status, alcohol drinking, healthy diet, leisure time physical activity, eGFR, family history of CVD, diabetes, hypertension, and hypercholesterolemia.

### 3.4 Threshold effects of CALLY index on CVD incidence and mortality in patients with CKD

**Figure 2:**
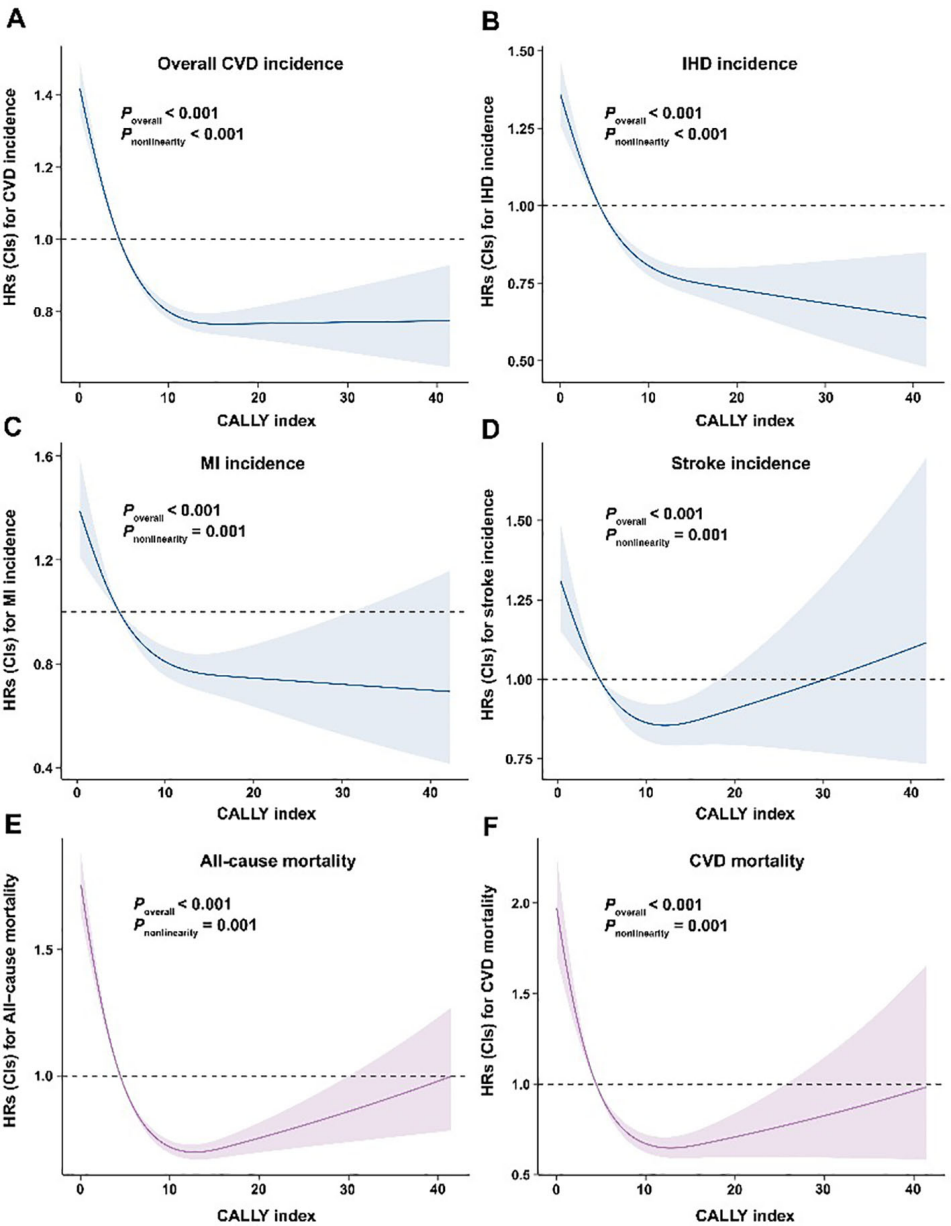
Dose-response association between CALLY index and CVD incidence and all-cause and CVD-specific mortality. **(A)** CVD incidence; **(B)** ischemic heart disease; **(C)** myocardial infarction; **(D)** stroke; **(E)** all-cause mortality; **(F)** CVD-specific mortality. Model was adjusted for age, sex, ethnicity, educational attainment, and socioeconomic deprivation, body mass index, smoking status, alcohol drinking, healthy diet, leisure time physical activity, eGFR, family history of CVD, diabetes, hypertension, and hypercholesterolemia.

The RCS curves showed the significant L-shaped dose-response relationships between CALLY index and the CVD incidence (including overall CVD, IHD, MI, and stroke), all-cause and CVD-specific mortality (all *P_overall_* < 0.001; *P_nonlinear_* < 0.05) (**Figures 2A-F**). To evaluate the nonlinear associations between the CALLY index and corresponding outcomes, we further conducted segmented Cox proportional hazards regression analyses on both sides of the inflection point (**Table 4**). The results demonstrated a significant inverse relationship between the CALLY index and the risks of incident overall CVD, IHD, MI, stroke, as well as all-cause and CVD-specific mortality below the inflection point. However, beyond this threshold, no statistically significant associations were observed between the CALLY index and either CVD incidence or mortality outcomes.

**Table 4:** Threshold effect analyses of CALLY index with CVD incidence and all-cause and CVD-specific mortality in CKD patients.

Outcomes	Before inflection point	After inflection point	P for log-likelihood ratio
CALLY index			
Overall CVD incidence			
Inflection point	2.94		
HR (95% CI)	0.85 (0.82, 0.87)	1.00 (1.00, 1.00)	<0.001
MI incidence			
Inflection point	5.28		
HR (95% CI)	0.92 (0.89, 0.96)	0.99 (0.98, 1.00)	0.001
IHD incidence			
Inflection point	4.88		
HR (95% CI)	0.92 (0.90, 0.94)	1.00 (0.99, 1.00)	<0.001
Stroke incidence			
Inflection point	3.82		
HR (95% CI)	0.89 (0.85, 0.94)	1.00 (0.99, 1.01)	<0.001
All-cause mortality			
Inflection point	5.06		
HR (95% CI)	0.85 (0.84, 0.87)	1.00 (1.00, 1.01)	<0.001
CVD mortality			
Inflection point	6.14		
HR (95% CI)	0.86 (0.84, 0.89)	1.01 (1.00, 1.01)	<0.001

Models were adjusted for age, sex, ethnicity, educational attainment, socioeconomic deprivation, body mass index, smoking status, alcohol drinking, healthy diet, leisure time physical activity, eGFR, family history of CVD, diabetes, hypertension, and hypercholesterolemia.

### 3.5 Subgroup and sensitivity analyses

Sensitivity analysis results showed that the associations remained robust after excluding participants with < 2 years of follow-up (**Supplementary Tables S2**, **S3**). Furthermore, sensitivity analysis excluding ethnicity from the covariates showed that the associations of the CALLY Index with CVD incidence and all-cause and CVD-specific mortality did not appreciably change (**Supplementary Table S4**). The subgroup analyses demonstrated consistent inverse associations between higher CALLY index and decreased risk of CVD incidence (including IHD, MI, and stroke) and mortality (all-cause and CVD-specific) across all predefined subgroups of CKD patients. No significant interactions were found for most subgroups (P for interaction >0.05), except for age in MI incidence and smoking status in MI and IHD incidence, where younger individuals and past/current smokers exhibited stronger inverse associations. Furthermore, patients with lower eGFR exhibited slightly stronger inverse associations between CALLY index and all-cause mortality (**Supplementary Tables S5**-**S10**).

### 3.6 Predictive value of CALLY index and other inflammatory biomarkers on CVD incidence and mortality

To evaluate and compare the independent predictive capacity of CALLY and other inflammatory biomarkers, including SII, MLR, and NLR, we calculated the Harrell’s C-index for CVD incidence and all-cause and CVD-specific mortality, as shown in **Supplementary Figure S1**. The results showed that CALLY exhibited a higher Harrell’s C-index for all outcomes compared to the other inflammatory biomarkers.

## 4 Discussion

Utilizing data from the UK Biobank, our study provides novel evidence supporting the CALLY index as an independent predictor of adverse cardiovascular outcomes in CKD patients. We observed significant inverse associations between the CALLY index and CVD incidence, all-cause mortality, and CVD-specific mortality, with higher index values consistently correlating with lower risks of these endpoints. Importantly, these associations remained robust even after extensive adjustment for key confounders, including lifestyle factors (diet, physical activity, smoking, and alcohol consumption) and renal function (eGFR). These findings suggest that the CALLY index, integrating inflammatory, nutritional, and immunological markers, may serve as a valuable tool for risk stratification and mortality prediction in CKD patients, potentially enhancing early intervention strategies for this high-risk population.

To our knowledge, epidemiological evidence regarding the associations of CALLY index with incidence of CVD and all-cause and CVD-specific mortality remain unclear in patients with CKD. Based on a prospective cohort design, our findings provide the first evidence that CALLY index as an independent predictor of CVD incidence and prognosis in CKD patients, underscoring its potential for integration into clinical prediction models to enable personalized risk management. Notably, a prior cross-sectional analyses included 8,146 participants from the National Health and Nutrition Examination Survey (NHANES) database showed that the CALLY index may be a potential indicator for early identification of individuals at higher risk of stroke in hypertensive patients (22). Another study found the inverse relationship between the CALLY index and angina in United States adults (33). Interestingly, emerging evidence has demonstrated the significant negative associations between the CALLY index and risk of PAD (20), osteoarthritis (34), depression (21), and retinopathy (33). However, the aforementioned studies were primarily limited to cross-sectional designs with relatively small sample sizes. In addition, studies in the field of disease prognosis assessment have shown that CALLY index can be used as an independent prognostic factor for patients with COPD (35), acute ischemic stroke (36), CVD (37), sepsis (38), and multiple types of cancers (11, 23, 39, 40). By contrast, its potential utility in CKD populations remains to be fully elucidated, with no dedicated studies currently available in this specific patient cohort.

CVD risk emerges early in CKD and escalates progressively with disease severity (4, 41). CKD predisposes patients to diverse CVD subtypes, including IHD (42), MI (43), and stroke (44), driven by a pathophysiological triad of chronic inflammation, malnutrition, and immune dysfunction that synergistically accelerates CVD progression (45). Central to this process is low-grade microinflammation, characterized by elevated CRP-a hepatic acute-phase reactant upregulated by pro-inflammatory cytokines such as IL-6 (46). In CKD, uremic toxin accumulation, oxidative stress, and gut dysbiosis perpetuate inflammatory pathway activation (47), increasing circulating CRP and IL-6. These mediators exacerbate endothelial dysfunction, atherosclerotic plaque instability (48, 49), and ultimately, thrombotic events such as MI and stroke (50, 51). Concurrently, lymphopenia, a marker of immunosuppression, reflects immunosenescence and malnutrition (52). Emerging therapies like finerenone, a nonsteroidal mineralocorticoid receptor antagonist (MRA), mitigate inflammation and renal injury while preserving potassium homeostasis, yet may inadvertently modulate T-cell function via the IL-17/IL-23 axis (53). Similarly, systemic immune-inflammation (e.g., elevated SII) exacerbates renal fibrosis by suppressing the anti-aging protein α-Klotho (54), further compounding CVD risk. Hypoalbuminemia, prevalent in CKD, reflecting both protein-energy wasting and inflammatory catabolism. Depleted albumin impairs antioxidant, anticoagulant, and endothelial protective functions, promoting coronary stenosis and thrombosis (55). Given the complex pathophysiology of CVD, reliance on a single biomarker is likely insufficient to comprehensively capture multidimensional health risk of patients. Instead, integrated indices like the CALLY index, combining CRP (inflammation), albumin (nutrition), and lymphocytes (immunity) (36, 56), offer a holistic assessment of this pathogenic triad (37, 49), thereby overcoming the limitations of conventional single-parameter biomarkers. Higher CALLY values signify optimal inflammatory control and immune-nutritional homeostasis, supporting its utility in risk stratification and personalized CVD prevention.

In the present study, the RCS analyses revealed significant nonlinear, L-shaped associations between the CALLY index and all examined clinical endpoints (CVD incidence, all-cause mortality, and CVD-specific mortality). This nonlinear pattern indicates a saturation effect, where excessively low CALLY index values are associated with increased risk of CVD events, while intermediate and high levels confer optimal protection. Previous studies have also reported similar L-shaped associations between the CALLY index and various health outcomes, including all-cause mortality in rheumatoid arthritis patients (57), stroke (58), and angina pectoris risk (59). The observed L-shaped relationships may be attributed to the role of CALLY index as an integrative biomarker of inflammation, nutrition, and immune homeostasis. At low CALLY levels, the concomitant presence of chronic inflammation, malnutrition, and immune dysregulation likely synergistically promotes the CVD pathogenesis. As the CALLY index increases, the mitigation of these pathological processes may explain the plateau in risk reduction at intermediate-to-high levels, where further increments no longer confer additional benefits (59). This saturation effect may align with biological thresholds beyond which inflammation is adequately controlled, nutritional reserves are sufficient, and immune responses reach equilibrium. Notably, the consistency of L-shaped associations across diverse endpoints and populations underscores the broad utility of CALLY index in risk stratification, while its non-linearity highlights the need for targeted interventions in individuals with critically low values. The inflection point of this curve provides a practical threshold for risk stratification. The primary clinical utility of this index lies in risk stratification. Moreover, compared with traditional risk scores based on demographic and metabolic parameters, the CALLY index offers a direct and complementary measure that reflects the pathological interplay of inflammation, nutrition, and immunity relevant to CKD. Future research should focus on prospectively validating this specific threshold in independent cohorts and exploring whether integrating the CALLY index with established risk models enhances prognostic accuracy for CKD patients.

Our study has several strengths. Firstly, the inclusion of participants from the UK Biobank based on a nationally-representative population provided a relatively large sample size and extended follow-up period, ensuring robust statistical power for detecting clinically meaningful associations and mitigating risks of selection bias. Secondly, we comprehensively adjusted for key confounders including sociodemographic characteristics, detailed lifestyle factors, dietary, anthropometric measures, and complete medical history, which substantially reduces potential confounding effects. Thirdly, to examine the robustness of our findings, we comprehensively conducted sensitivity and subgroup analyses and found that the results were largely unchanged. Moreover, our study employed RCS analysis to comprehensively characterize the complex dose-response relationships between the CALLY index and incidence of CVD and all-cause and CVD-specific mortality, and identified precise inflection points where CALLY index values demonstrated maximum protective effects against outcomes through further application of two-segmented Cox proportional hazard models. This methodological innovation may contribute to determining clinically meaningful threshold values that may inform future risk stratification protocols.

However, several limitations warrant consideration when interpreting our results. Firstly, the single measurement of CRP, albumin, and lymphocyte may not adequately capture their dynamic biological variations. Future longitudinal studies incorporating repeated assessments would be valuable to better characterize the temporal trajectory of the CALLY index and its association with cardiovascular events and mortality in patients with CKD, thereby improving causal inference. Secondly, as an observational study, our findings demonstrate associations but cannot establish causality. Despite extensive covariate adjustment, potential confounding by unmeasured factors such as environmental exposures, genetic predispositions, or medication use (e.g., statins, anti-inflammatory agents, dialysis therapy) remain possible. Thirdly, and importantly, the predominantly White European ancestry of UK Biobank participants significantly limits the generalizability of our findings. Differences in genetic background, lifestyle, comorbidities, and the prevalence and etiology of CKD across ethnic groups may influence the association between the CALLY index and cardiovascular outcomes. Therefore, our results require explicit validation in more ethnically and geographically diverse populations, before they can be considered broadly applicable. Future studies should elucidate associations between the CALLY index and underlying pathophysiological pathways of CVD in CKD models, while designing and implementing CALLY-directed precision intervention trials (nutritional/anti-inflammatory regimens) to develop prognostic risk reduction strategies for CVD in CKD populations.

## 5 Conclusions

In this large prospective cohort study of patients with CKD, we demonstrated that a moderate-to-high CALLY index was significantly associated with a reduced risk of CVD events-including IHD, MI, and stroke, as well as lower all-cause and CVD-specific mortality. In addition, our findings suggest that the CALLY index shows a stronger association with cardiovascular risk than conventional inflammatory biomarkers, independent of traditional risk factors. Importantly, our results highlight the clinical utility of the CALLY index as an objective and integrative tool for early CVD risk stratification in CKD patients.

## Data Availability

The original contributions presented in the study are included in the article/**Supplementary Material**, further inquiries can be directed to the corresponding author.
