# Supplementary material for: Association between the C-reactive protein-albumin-lymphocyte index and cardiovascular incidence and mortality among patients with chronic kidney disease: a prospective study
Source: Front Immunol. 2026 Feb 6;17:1729647. doi: 10.3389/fimmu.2026.1729647 (PMC12920216; doi:10.3389/fimmu.2026.1729647)
Supplement: Supplementary file 1 [file DataSheet1.docx]

**Supplementary materials**

**Supplementary Table S1.** **Variance inflation factors for all variables in the regression model**

**Supplementary Table S2. Associations between CALLY index with CVD incidence** i**n CKD patients excluding those with less than 2 years of follow-up**

**Supplementary Table S3. Associations between CALLY index and all-cause and CVD-specifi**c **mortality** i**n CKD patients excluding those with less than 2 years of follow-up**

**Supplementary Table S4. Sensitivity analysis excluding** ethnicity **from the covariates: associations of the CALLY Index with CVD incidence and all-cause and CVD-specific mortality in CKD patients**

**Supplementary Table S5. Subgroup analysis of associations between CALLY index and overall CVD incidence** i**n CKD patients**

**Supplementary Table S6. Subgroup analysis of associations between CALLY index and IHD incidence** i**n CKD patients**

**Supplementary Table S7. Subgroup analysis of associations between CALLY index and MI incidence** i**n CKD patients**

**Supplementary Table S8. Subgroup analysis of associations between CALLY index and stroke incidence** i**n CKD patients**

**Supplementary Table S9. Subgroup analysis of associations between CALLY index and** all-cause mortality i**n CKD patients**

**Supplementary Table S10. Subgroup analysis of associations between CALLY index and** CVD-specific mortality i**n CKD patients**

**Supplementary Figure S1.** The independent predictive capacity of CALLY and other inflammatory biomarkers for CVD incidence and all-cause and CVD-specific mortality i**n CKD patients**

**Supplementary Table S1.** **Variance inflation factors for all variables in the regression model**

|  | **Variance inflation factors** |
| --- | --- |
| **CALLY index** | 1 |
| **Age** | 1.1 |
| **Sex** | 1 |
| Educational attainment | 1.2 |
| eGFR | 9.3 |
| Ethnicity | 9.7 |
| Socioeconomic deprivation | 1 |
| Body mass index | 1 |
| Alcohol drinking | 2.1 |
| Smoking status | 1.4 |
| Diabetes | 1.5 |
| Hypertension | 2.5 |
| Healthy diet | 1.1 |
| Leisure time physical activity | 1.1 |
| Hypercholesterolemia | 1 |
| Family history of CVD | 1 |

**Supplementary Table S2.** **Associations between CALLY index with CVD incidence** i**n CKD patients excluding those with less than 2 years of follow-up**

|  | Quartiles of CALLY index | | | | P for trend | Per 1-SD increase in CALLY index |
| --- | --- | --- | --- | --- | --- | --- |
|  | Q1 | Q2 | Q3 | Q4 |  |  |
| CVD incidence |  |  |  |  |  |  |
| Events | 1891(35.29) | 1633 (29.86) | 1427 (25.96) | 1255 (22.51) |  |  |
| Crude | 1.00 (Ref.) | 0.80 (0.75, 0.85) | 0.67 (0.63, 0.72) | 0.57 (0.53, 0.61) | <0.001 | 0.81 (0.79, 0.84) |
| Model 1 | 1.00 (Ref.) | 0.79 (0.74, 0.84) | 0.68 (0.63, 0.72) | 0.63 (0.58, 0.67) | <0.001 | 0.87 (0.84, 0.90) |
| Model 2 | 1.00 (Ref.) | 0.82 (0.77, 0.88) | 0.74 (0.69, 0.79) | 0.72 (0.67, 0.77) | <0.001 | 0.92 (0.89, 0.96) |
| IHD incidence |  |  |  |  |  |  |
| Events | 902 (16.33) | 769 (13.80) | 629 (11.23) | 549 (9.73) |  |  |
| Crude | 1.00 (Ref.) | 0.81 (0.73, 0.89) | 0.64 (0.58, 0.71) | 0.55 (0.49, 0.61) | <0.001 | 0.78 (0.73, 0.82) |
| Model 1 | 1.00 (Ref.) | 0.80 (0.72, 0.88) | 0.65 (0.59, 0.72) | 0.61 (0.55, 0.68) | <0.001 | 0.83 (0.79, 0.88) |
| Model 2 | 1.00 (Ref.) | 0.84 (0.76, 0.93) | 0.72 (0.65, 0.80) | 0.70 (0.62, 0.78) | <0.001 | 0.89 (0.84, 0.94) |
| MI incidence |  |  |  |  |  |  |
| Events | 283 (5.06) | 264 (4.68) | 193 (3.40) | 172 (3.02) |  |  |
| Crude | 1.00 (Ref.) | 0.89 (0.75, 1.05) | 0.63 (0.53, 0.76) | 0.56 (0.46, 0.68) | <0.001 | 0.75 (0.67, 0.83) |
| Model 1 | 1.00 (Ref.) | 0.88 (0.74, 1.04) | 0.65 (0.54, 0.78) | 0.63 (0.52, 0.76) | <0.001 | 0.81 (0.73, 0.90) |
| Model 2 | 1.00 (Ref.) | 0.93 (0.78, 1.10) | 0.71 (0.59, 0.86) | 0.70 (0.58, 0.86) | <0.001 | 0.85 (0.77, 0.94) |
| Stroke incidence |  |  |  |  |  |  |
| Events | 301 (5.39) | 271 (4.80) | 246 (4.33) | 223 (3.92) |  |  |
| Crude | 1.00 (Ref.) | 0.86 (0.73, 1.01) | 0.76 (0.64, 0.90) | 0.68 (0.58, 0.81) | <0.001 | 0.89 (0.82, 0.96) |
| Model 1 | 1.00 (Ref.) | 0.85 (0.72, 1.00) | 0.77 (0.65, 0.91) | 0.75 (0.63, 0.90) | <0.001 | 0.94 (0.87, 1.02) |
| Model 2 | 1.00 (Ref.) | 0.85 (0.72, 1.01) | 0.78 (0.66, 0.93) | 0.75 (0.63, 0.90) | <0.001 | 0.94 (0.87, 1.02) |

Model 1 was adjusted age, sex, ethnicity, educational attainment, and socioeconomic deprivation; Model 2 was further adjusted for body mass index, smoking status, alcohol drinking, healthy diet, leisure time physical activity, eGFR, family history of CVD, diabetes, hypertension, and hypercholesterolemia.

**Supplementary Table S3. Associations between CALLY index and all-cause and CVD-specifi**c **mortality** i**n CKD patients excluding those with less than 2 years of follow-up**

|  | Quartiles of CALLY index | | | |  |  |
| --- | --- | --- | --- | --- | --- | --- |
|  | Q1 | Q2 | Q3 | Q4 | P for trend | Per 1-SD increase in CALLY index |
| All-cause mortality |  |  |  |  |  |  |
| Events | 1416 (25.23) | 1071 (18.92) | 815 (14.31) | 728 (12.77) |  |  |
| Crude | 1.00 (Ref.) | 0.72 (0.66, 0.78) | 0.53 (0.49, 0.58) | 0.47 (0.43, 0.52) | <0.001 | 0.78 (0.75, 0.82) |
| Model 1 | 1.00 (Ref.) | 0.72 (0.66, 0.78) | 0.55 (0.50, 0.60) | 0.55 (0.51, 0.61) | <0.001 | 0.86 (0.82, 0.90) |
| Model 2 | 1.00 (Ref.) | 0.75 (0.69, 0.81) | 0.59 (0.54, 0.65) | 0.59 (0.53, 0.64) | <0.001 | 0.88 (0.84, 0.93) |
| CVD mortality |  |  |  |  |  |  |
| Events | 338 (6.02) | 271 (4.79) | 171 (3.00) | 158 (2.77) |  |  |
| Crude | 1.00 (Ref.) | 0.76 (0.65, 0.89) | 0.47 (0.39, 0.56) | 0.43 (0.36, 0.52) | <0.001 | 0.72 (0.64, 0.80) |
| Model 1 | 1.00 (Ref.) | 0.75 (0.64, 0.88) | 0.48 (0.40, 0.57) | 0.50 (0.41, 0.60) | <0.001 | 0.79 (0.71, 0.88) |
| Model 2 | 1.00 (Ref.) | 0.79 (0.68, 0.93) | 0.53 (0.44, 0.64) | 0.57 (0.46, 0.69) | <0.001 | 0.85 (0.76, 0.95) |

Model 1 was adjusted age, sex, ethnicity, educational attainment, and socioeconomic deprivation; Model 2 was further adjusted for body mass index, smoking status, alcohol drinking, healthy diet, leisure time physical activity, eGFR, family history of CVD, diabetes, hypertension, and hypercholesterolemia.

**Supplementary Table S4.** **Sensitivity analysis excluding** ethnicity **from the covariates: associations of the CALLY Index with CVD incidence and all-cause and CVD-specific mortality in CKD patients**

|  | Quartiles of CALLY index | | | | P for trend | Per 1-SD increase in CALLY index |
| --- | --- | --- | --- | --- | --- | --- |
|  | Q1 | Q2 | Q3 | Q4 |  |  |
| CVD incidence |  |  |  |  |  |  |
| Multivariate model | 1.00 (Ref.) | 0.81 (0.77, 0.87) | 0.74 (0.70, 0.79) | 0.70 (0.65, 0.75) | <0.001 | 0.91 (0.88, 0.94) |
| IHD incidence |  |  |  |  |  |  |
| Multivariate model | 1.00 (Ref.) | 0.86 (0.78, 0.94) | 0.75 (0.68, 0.83) | 0.70 (0.63, 0.78) | <0.001 | 0.89 (0.84, 0.93) |
| MI incidence |  |  |  |  |  |  |
| Multivariate model | 1.00 (Ref.) | 0.94 (0.80, 1.11) | 0.72 (0.60, 0.86) | 0.68 (0.57, 0.83) | <0.001 | 0.84 (0.76, 0.92) |
| Stroke incidence |  |  |  |  |  |  |
| Multivariate model | 1.00 (Ref.) | 0.85 (0.72, 0.99) | 0.77 (0.65, 0.91) | 0.74 (0.62, 0.89) | <0.001 | 0.94 (0.87, 1.02) |
| All-cause mortality |  |  |  |  |  |  |
| Multivariate model | 1.00 (Ref.) | 0.74 (0.68, 0.79) | 0.57 (0.52, 0.62) | 0.55 (0.50, 0.60) | <0.001 | 0.86 (0.82, 0.90) |
| CVD mortality |  |  |  |  |  |  |
| Multivariate model | 1.00 (Ref.) | 0.79 (0.68, 0.93) | 0.52 (0.43, 0.62) | 0.54 (0.44, 0.65) | <0.001 | 0.82 (0.74, 0.92) |

Multivariate model was adjusted age, sex, educational attainment, socioeconomic deprivation, body mass index, smoking status, alcohol drinking, healthy diet, leisure time physical activity, eGFR, family history of CVD, diabetes, hypertension, and hypercholesterolemia.

**Supplementary Table S5. Subgroup analysis of associations between CALLY index and overall CVD incidence** i**n CKD patients**

|  |  | Quartiles of CALLY index | | | |  | Per 1-SD increase  in CALLY index | P for  interaction |
| --- | --- | --- | --- | --- | --- | --- | --- | --- |
|  | Subgroup | Q1 | Q2 | Q3 | Q4 | P for trend |  |  |
| Age | < 60 years | 1.00 (Ref.) | 0.85 (0.76, 0.96) | 0.78 (0.69, 0.88) | 0.67 (0.59, 0.77) | <0.001 | 0.92 (0.87, 0.98) | 0.433 |
|  | ≥ 60 years | 1.00 (Ref.) | 0.80 (0.75, 0.86) | 0.72 (0.67, 0.78) | 0.69 (0.64, 0.76) | <0.001 | 0.90 (0.86, 0.94) |  |
| Sex | Male | 1.00 (Ref.) | 0.82 (0.75, 0.89) | 0.72 (0.66, 0.79) | 0.69 (0.63, 0.76) | <0.001 | 0.91 (0.87, 0.96) | 0.566 |
|  | Female | 1.00 (Ref.) | 0.80 (0.73, 0.88) | 0.76 (0.69, 0.84) | 0.70 (0.63, 0.79) | <0.001 | 0.92 (0.88, 0.97) |  |
| Qualifications | No relevant qualifications | 1.00 (Ref.) | 0.74 (0.65, 0.85) | 0.72 (0.63, 0.83) | 0.67 (0.58, 0.77) | <0.001 | 0.98 (0.93, 1.04) | 0.308 |
|  | College or University degree | 1.00 (Ref.) | 0.65 (0.52, 0.82) | 0.72 (0.57, 0.90) | 0.66 (0.51, 0.85) | 0.004 | 0.94 (0.84, 1.06) |  |
|  | Others | 1.00 (Ref.) | 0.85 (0.79, 0.91) | 0.74 (0.69, 0.80) | 0.70 (0.64, 0.76) | <0.001 | 0.87 (0.83, 0.91) |  |
| Townsend  deprivation index | Low | 1.00 (Ref.) | 0.80 (0.73, 0.88) | 0.77 (0.70, 0.85) | 0.69 (0.62, 0.77) | <0.001 | 0.92 (0.87, 0.96) | 0.532 |
|  | High | 1.00 (Ref.) | 0.82 (0.75, 0.89) | 0.71 (0.65, 0.78) | 0.70 (0.63, 0.77) | <0.001 | 0.91 (0.87, 0.96) |  |
| Body mass index | < 30 kg/m^2^ | 1.00 (Ref.) | 0.83 (0.76, 0.91) | 0.75 (0.68, 0.81) | 0.72 (0.66, 0.79) | <0.001 | 0.94 (0.90, 0.97) | 0.879 |
|  | ≥ 30 kg/m^2^ | 1.00 (Ref.) | 0.81 (0.74, 0.88) | 0.75 (0.68, 0.83) | 0.69 (0.60, 0.78) | <0.001 | 0.87 (0.81, 0.94) |  |
| Smoking status | Never | 1.00 (Ref.) | 0.86 (0.78, 0.95) | 0.81 (0.73, 0.89) | 0.76 (0.69, 0.85) | <0.001 | 0.95 (0.90, 0.99) | 0.060 |
|  | Past or current | 1.00 (Ref.) | 0.78 (0.72, 0.85) | 0.69 (0.63, 0.75) | 0.64 (0.58, 0.71) | <0.001 | 0.88 (0.83, 0.92) |  |
| Alcohol | Never | 1.00 (Ref.) | 0.93 (0.73, 1.20) | 0.73 (0.56, 0.97) | 0.89 (0.66, 1.19) | 0.1435 | 0.97 (0.84, 1.13) | 0.277 |
|  | Past or current | 1.00 (Ref.) | 0.81 (0.76, 0.86) | 0.74 (0.69, 0.79) | 0.68 (0.63, 0.74) | <0.001 | 0.91 (0.87, 0.94) |  |
| Leisure time  physical activity | < 500 MET mins/week | 1.00 (Ref.) | 0.83 (0.75, 0.92) | 0.72 (0.65, 0.80) | 0.67 (0.59, 0.75) | <0.001 | 0.91 (0.86, 0.97) | 0.876 |
|  | ≥ 500 MET mins/week | 1.00 (Ref.) | 0.81 (0.74, 0.90) | 0.74 (0.67, 0.81) | 0.70 (0.63, 0.77) | <0.001 | 0.91 (0.87, 0.95) |  |
| Healthy diet | No | 1.00 (Ref.) | 0.79 (0.73, 0.85) | 0.73 (0.68, 0.79) | 0.69 (0.64, 0.75) | <0.001 | 0.91 (0.87, 0.95) | 0.130 |
|  | Yes | 1.00 (Ref.) | 0.96 (0.83, 1.12) | 0.79 (0.67, 0.94) | 0.72 (0.61, 0.86) | <0.001 | 0.92 (0.86, 1.00) |  |
| eGFR | Low | 1.00 (Ref.) | 0.79 (0.73, 0.86) | 0.71 (0.65, 0.77) | 0.67 (0.61, 0.74) | <0.001 | 0.90 (0.86, 0.95) | 0.378 |
|  | High | 1.00 (Ref.) | 0.84 (0.77, 0.93) | 0.80 (0.72, 0.88) | 0.73 (0.66, 0.82) | <0.001 | 0.92 (0.88, 0.97) |  |
| Family history  of CVD | No | 1.00 (Ref.) | 0.81 (0.75, 0.88) | 0.75 (0.69, 0.81) | 0.72 (0.65, 0.79) | <0.001 | 0.93 (0.89, 0.97) | 0.729 |
|  | Yes | 1.00 (Ref.) | 0.82 (0.74, 0.90) | 0.73 (0.66, 0.81) | 0.67 (0.60, 0.75) | <0.001 | 0.89 (0.85, 0.94) |  |
| Diabetes | No | 1.00 (Ref.) | 0.80 (0.75, 0.86) | 0.73 (0.68, 0.79) | 0.69 (0.64, 0.75) | <0.001 | 0.90 (0.87, 0.94) | 0.720 |
|  | Yes | 1.00 (Ref.) | 0.87 (0.75, 1.01) | 0.79 (0.68, 0.93) | 0.73 (0.62, 0.86) | <0.001 | 0.95 (0.88, 1.02) |  |
| Hypertension | No | 1.00 (Ref.) | 0.85 (0.77, 0.94) | 0.77 (0.70, 0.86) | 0.71 (0.63, 0.79) | <0.001 | 0.94 (0.89, 0.98) | 0.734 |
|  | Yes | 1.00 (Ref.) | 0.80 (0.73, 0.86) | 0.73 (0.67, 0.79) | 0.69 (0.63, 0.76) | <0.001 | 0.89 (0.85, 0.94) |  |
| Hyperlipidemia | No | 1.00 (Ref.) | 0.80 (0.75, 0.86) | 0.73 (0.68, 0.79) | 0.69 (0.64, 0.75) | <0.001 | 0.90 (0.86, 0.93) | 0.897 |
|  | Yes | 1.00 (Ref.) | 0.85 (0.75, 0.97) | 0.76 (0.67, 0.87) | 0.71 (0.62, 0.82) | <0.001 | 0.95 (0.89, 1.01) |  |

Model 1 was adjusted age, sex, ethnicity, educational attainment, and socioeconomic deprivation, body mass index, smoking status, alcohol drinking, healthy diet, leisure time physical activity, eGFR, family history of CVD, diabetes, hypertension, and hypercholesterolemia.

**Supplementary Table S6. Subgroup analysis of associations between CALLY index and IHD incidence** i**n CKD patients**

|  |  | Quartiles of CALLY index | | | |  | Per 1-SD increase in CALLY index | P for interaction |
| --- | --- | --- | --- | --- | --- | --- | --- | --- |
|  | Subgroup | Q1 | Q2 | Q3 | Q4 | P for trend |  |  |
| Age | < 60 years | 1.00 (Ref.) | 1.01 (0.85, 1.20) | 0.84 (0.70, 1.02) | 0.66 (0.54, 0.80) | <0.001 | 0.83 (0.76, 0.92) | 0.023 |
|  | ≥ 60 years | 1.00 (Ref.) | 0.80 (0.72, 0.89) | 0.71 (0.63, 0.80) | 0.71 (0.63, 0.80) | <0.001 | 0.90 (0.85, 0.96) |  |
| Sex | Male | 1.00 (Ref.) | 0.86 (0.77, 0.97) | 0.74 (0.66, 0.84) | 0.68 (0.60, 0.78) | <0.001 | 0.86 (0.80, 0.92) | 0.784 |
|  | Female | 1.00 (Ref.) | 0.83 (0.71, 0.96) | 0.75 (0.64, 0.88) | 0.73 (0.61, 0.87) | <0.001 | 0.93 (0.86, 1.01) |  |
| Qualifications | No relevant qualifications | 1.00 (Ref.) | 0.76 (0.62, 0.93) | 0.71 (0.58, 0.88) | 0.62 (0.50, 0.77) | <0.001 | 0.91 (0.83, 1.00) | 0.744 |
|  | College or University degree | 1.00 (Ref.) | 0.76 (0.54, 1.08) | 0.80 (0.57, 1.13) | 0.70 (0.48, 1.03) | 0.1039 | 0.81 (0.65, 1.00) |  |
|  | Others | 1.00 (Ref.) | 0.89 (0.80, 0.99) | 0.75 (0.67, 0.85) | 0.72 (0.63, 0.82) | <0.001 | 0.87 (0.82, 0.94) |  |
| Townsend deprivation index | Low | 1.00 (Ref.) | 0.85 (0.74, 0.98) | 0.78 (0.68, 0.90) | 0.71 (0.61, 0.83) | <0.001 | 0.88 (0.82, 0.95) | 0.888 |
|  | High | 1.00 (Ref.) | 0.85 (0.76, 0.96) | 0.73 (0.63, 0.83) | 0.70 (0.60, 0.81) | <0.001 | 0.89 (0.83, 0.96) |  |
| Body mass index | < 30 kg/m^2^ | 1.00 (Ref.) | 0.87 (0.76, 0.99) | 0.70 (0.62, 0.81) | 0.70 (0.61, 0.80) | <0.001 | 0.90 (0.84, 0.95) | 0.267 |
|  | ≥ 30 kg/m^2^ | 1.00 (Ref.) | 0.84 (0.74, 0.96) | 0.83 (0.72, 0.96) | 0.71 (0.59, 0.86) | <0.001 | 0.87 (0.78, 0.97) |  |
| Smoking status | Never | 1.00 (Ref.) | 0.91 (0.78, 1.05) | 0.85 (0.73, 0.99) | 0.83 (0.71, 0.98) | 0.0178 | 0.96 (0.90, 1.03) | 0.033 |
|  | Past or current | 1.00 (Ref.) | 0.83 (0.74, 0.93) | 0.68 (0.60, 0.78) | 0.61 (0.53, 0.71) | <0.001 | 0.81 (0.75, 0.87) |  |
| Alcohol | Never | 1.00 (Ref.) | 0.89 (0.63, 1.26) | 0.69 (0.47, 1.01) | 0.74 (0.49, 1.12) | 0.0565 | 0.95 (0.77, 1.17) | 0.903 |
|  | Past or current | 1.00 (Ref.) | 0.85 (0.78, 0.94) | 0.75 (0.68, 0.83) | 0.69 (0.62, 0.78) | <0.001 | 0.88 (0.83, 0.93) |  |
| Leisure time physical activity | < 500 MET mins/week | 1.00 (Ref.) | 0.84 (0.73, 0.97) | 0.73 (0.62, 0.85) | 0.65 (0.55, 0.78) | <0.001 | 0.84 (0.76, 0.92) | 0.922 |
|  | ≥ 500 MET mins/week | 1.00 (Ref.) | 0.89 (0.77, 1.02) | 0.74 (0.64, 0.85) | 0.70 (0.60, 0.82) | <0.001 | 0.89 (0.83, 0.96) |  |
| Healthy diet | No | 1.00 (Ref.) | 0.84 (0.76, 0.94) | 0.78 (0.70, 0.87) | 0.71 (0.63, 0.80) | <0.001 | 0.88 (0.83, 0.94) | 0.385 |
|  | Yes | 1.00 (Ref.) | 0.88 (0.71, 1.09) | 0.68 (0.53, 0.86) | 0.60 (0.47, 0.78) | <0.001 | 0.86 (0.76, 0.97) |  |
| eGFR | Low | 1.00 (Ref.) | 0.80 (0.71, 0.90) | 0.74 (0.65, 0.84) | 0.68 (0.59, 0.79) | <0.001 | 0.89 (0.83, 0.96) | 0.554 |
|  | High | 1.00 (Ref.) | 0.92 (0.80, 1.05) | 0.77 (0.66, 0.90) | 0.73 (0.62, 0.85) | <0.001 | 0.87 (0.81, 0.94) |  |
| Family history of CVD | No | 1.00 (Ref.) | 0.87 (0.77, 0.98) | 0.76 (0.67, 0.87) | 0.75 (0.65, 0.86) | <0.001 | 0.90 (0.84, 0.96) | 0.546 |
|  | Yes | 1.00 (Ref.) | 0.82 (0.71, 0.95) | 0.72 (0.62, 0.84) | 0.64 (0.54, 0.75) | <0.001 | 0.87 (0.80, 0.94) |  |
| Diabetes | No | 1.00 (Ref.) | 0.86 (0.78, 0.96) | 0.74 (0.66, 0.83) | 0.70 (0.62, 0.79) | <0.001 | 0.86 (0.81, 0.92) | 0.917 |
|  | Yes | 1.00 (Ref.) | 0.85 (0.70, 1.03) | 0.79 (0.65, 0.97) | 0.72 (0.59, 0.90) | 0.002 | 0.94 (0.86, 1.03) |  |
| Hypertension | No | 1.00 (Ref.) | 0.94 (0.81, 1.10) | 0.77 (0.66, 0.91) | 0.72 (0.61, 0.85) | <0.001 | 0.88 (0.81, 0.96) | 0.416 |
|  | Yes | 1.00 (Ref.) | 0.81 (0.72, 0.91) | 0.74 (0.66, 0.84) | 0.70 (0.61, 0.80) | <0.001 | 0.89 (0.83, 0.95) |  |
| Hyperlipidemia | No | 1.00 (Ref.) | 0.86 (0.78, 0.96) | 0.76 (0.68, 0.86) | 0.68 (0.60, 0.78) | <0.001 | 0.86 (0.80, 0.92) | 0.818 |
|  | Yes | 1.00 (Ref.) | 0.85 (0.70, 1.02) | 0.72 (0.60, 0.87) | 0.73 (0.60, 0.89) | <0.001 | 0.93 (0.85, 1.01) |  |

Model 1 was adjusted age, sex, ethnicity, educational attainment, and socioeconomic deprivation, body mass index, smoking status, alcohol drinking, healthy diet, leisure time physical activity, eGFR, family history of CVD, diabetes, hypertension, and hypercholesterolemia.

**Supplementary Table S7. Subgroup analysis of associations between CALLY index and MI incidence** i**n CKD patients**

|  |  | Quartiles of CALLY index | | | |  | Per 1-SD increase in CALLY index | P for interaction |
| --- | --- | --- | --- | --- | --- | --- | --- | --- |
|  | Subgroup | Q1 | Q2 | Q3 | Q4 | P for trend |  |  |
| Age | < 60 years | 1.00 (Ref.) | 1.41 (1.05, 1.88) | 0.81 (0.58, 1.13) | 0.64 (0.44, 0.91) | 0.001 | 0.68 (0.55, 0.83) | 0.001 |
|  | ≥ 60 years | 1.00 (Ref.) | 0.78 (0.64, 0.95) | 0.68 (0.55, 0.84) | 0.70 (0.56, 0.88) | <0.001 | 0.90 (0.80, 1.01) |  |
| Sex | Male | 1.00 (Ref.) | 0.94 (0.77, 1.14) | 0.71 (0.57, 0.88) | 0.64 (0.51, 0.82) | <0.001 | 0.79 (0.69, 0.90) | 0.764 |
|  | Female | 1.00 (Ref.) | 0.94 (0.71, 1.24) | 0.70 (0.52, 0.96) | 0.77 (0.55, 1.06) | 0.0328 | 0.92 (0.79, 1.07) |  |
| Qualifications | No relevant qualifications | 1.00 (Ref.) | 0.87 (0.60, 1.26) | 0.74 (0.50, 1.09) | 0.55 (0.35, 0.84) | 0.0046 | 0.75 (0.60, 0.95) | 0.807 |
|  | College or University degree | 1.00 (Ref.) | 0.85 (0.46, 1.58) | 0.75 (0.40, 1.41) | 0.71 (0.35, 1.43) | 0.2878 | 0.82 (0.56, 1.22) |  |
|  | Others | 1.00 (Ref.) | 1.00 (0.83, 1.21) | 0.70 (0.57, 0.88) | 0.75 (0.60, 0.95) | <0.001 | 0.87 (0.78, 0.98) |  |
| Townsend deprivation index | Low | 1.00 (Ref.) | 0.89 (0.69, 1.14) | 0.74 (0.57, 0.96) | 0.75 (0.57, 0.99) | 0.0156 | 0.87 (0.76, 1.00) | 0.561 |
|  | High | 1.00 (Ref.) | 0.98 (0.80, 1.21) | 0.69 (0.54, 0.89) | 0.63 (0.48, 0.82) | <0.001 | 0.81 (0.70, 0.94) |  |
| Body mass index | < 30 kg/m^2^ | 1.00 (Ref.) | 0.92 (0.73, 1.16) | 0.69 (0.55, 0.88) | 0.66 (0.52, 0.83) | <0.001 | 0.87 (0.77, 0.97) | 0.780 |
|  | ≥ 30 kg/m^2^ | 1.00 (Ref.) | 0.96 (0.77, 1.22) | 0.76 (0.57, 1.00) | 0.81 (0.58, 1.13) | 0.0523 | 0.79 (0.63, 0.99) |  |
| Smoking status | Never | 1.00 (Ref.) | 1.04 (0.79, 1.35) | 0.81 (0.61, 1.08) | 0.98 (0.74, 1.32) | 0.5102 | 0.96 (0.85, 1.09) | 0.013 |
|  | Past or current | 1.00 (Ref.) | 0.92 (0.75, 1.12) | 0.67 (0.54, 0.85) | 0.52 (0.40, 0.68) | <0.001 | 0.73 (0.63, 0.85) |  |
| Alcohol | Never | 1.00 (Ref.) | 1.08 (0.60, 1.96) | 0.59 (0.29, 1.22) | 0.74 (0.35, 1.53) | 0.1762 | 0.73 (0.46, 1.18) | 0.754 |
|  | Past or current | 1.00 (Ref.) | 0.93 (0.79, 1.10) | 0.72 (0.60, 0.86) | 0.67 (0.55, 0.82) | <0.001 | 0.84 (0.76, 0.93) |  |
| Leisure time physical activity | < 500 MET mins/week | 1.00 (Ref.) | 0.91 (0.70, 1.19) | 0.76 (0.57, 1.01) | 0.60 (0.43, 0.83) | 0.0012 | 0.69 (0.56, 0.84) | 0.659 |
|  | ≥ 500 MET mins/week | 1.00 (Ref.) | 0.94 (0.73, 1.19) | 0.65 (0.50, 0.85) | 0.68 (0.52, 0.89) | <0.001 | 0.92 (0.81, 1.04) |  |
| Healthy diet | No | 1.00 (Ref.) | 0.97 (0.81, 1.15) | 0.77 (0.64, 0.94) | 0.67 (0.54, 0.84) | <0.001 | 0.83 (0.74, 0.94) | 0.256 |
|  | Yes | 1.00 (Ref.) | 0.80 (0.53, 1.20) | 0.50 (0.31, 0.80) | 0.71 (0.45, 1.13) | 0.0398 | 0.88 (0.70, 1.11) |  |
| eGFR | Low | 1.00 (Ref.) | 0.83 (0.67, 1.02) | 0.74 (0.59, 0.93) | 0.68 (0.52, 0.87) | 0.0011 | 0.88 (0.77, 1.00) | 0.216 |
|  | High | 1.00 (Ref.) | 1.09 (0.86, 1.40) | 0.69 (0.52, 0.92) | 0.70 (0.52, 0.94) | 0.0016 | 0.80 (0.68, 0.93) |  |
| Family history of CVD | No | 1.00 (Ref.) | 0.98 (0.79, 1.21) | 0.77 (0.61, 0.97) | 0.76 (0.59, 0.98) | 0.0083 | 0.88 (0.78, 1.00) | 0.639 |
|  | Yes | 1.00 (Ref.) | 0.89 (0.70, 1.14) | 0.66 (0.50, 0.86) | 0.59 (0.44, 0.80) | <0.001 | 0.77 (0.65, 0.92) |  |
| Diabetes | No | 1.00 (Ref.) | 0.91 (0.75, 1.09) | 0.75 (0.61, 0.92) | 0.69 (0.55, 0.87) | <0.001 | 0.87 (0.78, 0.98) | 0.370 |
|  | Yes | 1.00 (Ref.) | 1.09 (0.78, 1.52) | 0.61 (0.42, 0.90) | 0.64 (0.43, 0.94) | 0.0024 | 0.74 (0.59, 0.91) |  |
| Hypertension | No | 1.00 (Ref.) | 1.03 (0.80, 1.33) | 0.73 (0.55, 0.98) | 0.65 (0.48, 0.89) | 0.0011 | 0.89 (0.77, 1.03) | 0.637 |
|  | Yes | 1.00 (Ref.) | 0.90 (0.73, 1.10) | 0.72 (0.57, 0.90) | 0.73 (0.57, 0.94) | 0.0021 | 0.80 (0.70, 0.93) |  |
| Hyperlipidemia | No | 1.00 (Ref.) | 0.92 (0.77, 1.11) | 0.77 (0.63, 0.94) | 0.69 (0.55, 0.87) | <0.001 | 0.84 (0.74, 0.94) | 0.427 |
|  | Yes | 1.00 (Ref.) | 1.05 (0.75, 1.46) | 0.61 (0.42, 0.89) | 0.70 (0.48, 1.02) | 0.0092 | 0.86 (0.71, 1.04) |  |

Model 1 was adjusted age, sex, ethnicity, educational attainment, and socioeconomic deprivation, body mass index, smoking status, alcohol drinking, healthy diet, leisure time physical activity, eGFR, family history of CVD, diabetes, hypertension, and hypercholesterolemia.

**Supplementary Table S8. Subgroup analysis of associations between CALLY index and stroke incidence** i**n CKD patients**

|  |  | Quartiles of CALLY index | | | |  | Per 1-SD increase in CALLY index | P for interaction |
| --- | --- | --- | --- | --- | --- | --- | --- | --- |
|  | Subgroup | Q1 | Q2 | Q3 | Q4 | P for trend |  |  |
| Age | < 60 years | 1.00 (Ref.) | 0.83 (0.61, 1.13) | 0.54 (0.38, 0.77) | 0.74 (0.53, 1.03) | 0.0166 | 1.03 (0.95, 1.12) | 0.076 |
|  | ≥ 60 years | 1.00 (Ref.) | 0.86 (0.72, 1.04) | 0.84 (0.70, 1.02) | 0.73 (0.59, 0.90) | 0.0037 | 0.87 (0.78, 0.96) |  |
| Sex | Male | 1.00 (Ref.) | 0.80 (0.65, 0.99) | 0.68 (0.54, 0.85) | 0.72 (0.57, 0.91) | 0.0015 | 0.97 (0.88, 1.07) | 0.784 |
|  | Female | 1.00 (Ref.) | 0.90 (0.71, 1.15) | 0.89 (0.70, 1.14) | 0.80 (0.61, 1.05) | 0.1163 | 0.92 (0.82, 1.04) |  |
| Qualifications | No relevant qualifications | 1.00 (Ref.) | 0.77 (0.54, 1.11) | 0.75 (0.52, 1.08) | 0.75 (0.52, 1.08) | 0.1473 | 1.05 (0.94, 1.18) | 0.977 |
|  | College or University degree | 1.00 (Ref.) | 0.94 (0.53, 1.70) | 0.91 (0.50, 1.65) | 0.97 (0.52, 1.78) | 0.8972 | 1.07 (0.94, 1.22) |  |
|  | Others | 1.00 (Ref.) | 0.85 (0.70, 1.02) | 0.76 (0.62, 0.92) | 0.72 (0.58, 0.89) | 0.001 | 0.84 (0.75, 0.95) |  |
| Townsend deprivation index | Low | 1.00 (Ref.) | 1.02 (0.81, 1.30) | 0.88 (0.69, 1.12) | 0.81 (0.62, 1.05) | 0.0581 | 0.91 (0.81, 1.03) | 0.197 |
|  | High | 1.00 (Ref.) | 0.73 (0.59, 0.90) | 0.70 (0.55, 0.87) | 0.70 (0.55, 0.89) | 0.0018 | 0.97 (0.88, 1.08) |  |
| Body mass index | < 30 kg/m^2^ | 1.00 (Ref.) | 0.86 (0.69, 1.07) | 0.88 (0.72, 1.09) | 0.80 (0.65, 1.00) | 0.0810 | 0.99 (0.92, 1.08) | 0.094 |
|  | ≥ 30 kg/m^2^ | 1.00 (Ref.) | 0.85 (0.67, 1.07) | 0.58 (0.43, 0.78) | 0.76 (0.55, 1.07) | 0.0027 | 0.80 (0.64, 1.01) |  |
| Smoking status | Never | 1.00 (Ref.) | 0.88 (0.69, 1.12) | 0.72 (0.56, 0.93) | 0.78 (0.60, 1.02) | 0.0262 | 0.97 (0.88, 1.09) | 0.558 |
|  | Past or current | 1.00 (Ref.) | 0.82 (0.66, 1.01) | 0.82 (0.66, 1.01) | 0.70 (0.55, 0.89) | 0.0049 | 0.91 (0.81, 1.02) |  |
| Alcohol | Never | 1.00 (Ref.) | 1.16 (0.62, 2.15) | 1.35 (0.71, 2.55) | 0.96 (0.45, 2.02) | 0.8518 | 0.99 (0.71, 1.37) | 0.355 |
|  | Past or current | 1.00 (Ref.) | 0.83 (0.70, 0.97) | 0.74 (0.62, 0.88) | 0.73 (0.61, 0.87) | <0.001 | 0.93 (0.86, 1.01) |  |
| Leisure time physical activity | < 500 MET mins/week | 1.00 (Ref.) | 0.76 (0.59, 0.98) | 0.78 (0.61, 1.01) | 0.54 (0.40, 0.73) | <0.001 | 0.85 (0.73, 0.99) | 0.010 |
|  | ≥ 500 MET mins/week | 1.00 (Ref.) | 0.98 (0.77, 1.25) | 0.80 (0.62, 1.03) | 0.98 (0.77, 1.26) | 0.5360 | 1.01 (0.92, 1.10) |  |
| Healthy diet | No | 1.00 (Ref.) | 0.76 (0.63, 0.92) | 0.74 (0.61, 0.89) | 0.74 (0.61, 0.90) | 0.0021 | 0.95 (0.86, 1.04) | 0.019 |
|  | Yes | 1.00 (Ref.) | 1.40 (0.97, 2.03) | 0.90 (0.60, 1.35) | 0.86 (0.57, 1.31) | 0.1535 | 0.95 (0.81, 1.12) |  |
| eGFR | Low | 1.00 (Ref.) | 0.81 (0.66, 1.00) | 0.73 (0.59, 0.91) | 0.68 (0.53, 0.87) | <0.001 | 0.92 (0.81, 1.03) | 0.682 |
|  | High | 1.00 (Ref.) | 0.92 (0.72, 1.18) | 0.83 (0.64, 1.07) | 0.84 (0.64, 1.09) | 0.1277 | 0.96 (0.87, 1.07) |  |
| Family history of CVD | No | 1.00 (Ref.) | 0.93 (0.76, 1.14) | 0.78 (0.63, 0.97) | 0.80 (0.64, 1.01) | 0.0221 | 0.96 (0.87, 1.06) | 0.400 |
|  | Yes | 1.00 (Ref.) | 0.72 (0.56, 0.93) | 0.74 (0.57, 0.96) | 0.65 (0.49, 0.86) | 0.0039 | 0.90 (0.79, 1.04) |  |
| Diabetes | No | 1.00 (Ref.) | 0.81 (0.67, 0.96) | 0.77 (0.64, 0.93) | 0.77 (0.63, 0.94) | 0.0057 | 0.92 (0.84, 1.01) | 0.316 |
|  | Yes | 1.00 (Ref.) | 1.09 (0.76, 1.55) | 0.80 (0.55, 1.17) | 0.71 (0.48, 1.05) | 0.0373 | 1.00 (0.87, 1.15) |  |
| Hypertension | No | 1.00 (Ref.) | 1.03 (0.80, 1.33) | 0.73 (0.55, 0.98) | 0.65 (0.48, 0.89) | 0.0011 | 0.89 (0.77, 1.03) | 0.045 |
|  | Yes | 1.00 (Ref.) | 0.90 (0.73, 1.10) | 0.72 (0.57, 0.90) | 0.73 (0.57, 0.94) | 0.0021 | 0.80 (0.70, 0.93) |  |
| Hyperlipidemia | No | 1.00 (Ref.) | 0.88 (0.74, 1.05) | 0.76 (0.63, 0.92) | 0.79 (0.65, 0.97) | 0.0066 | 0.96 (0.88, 1.05) | 0.298 |
|  | Yes | 1.00 (Ref.) | 0.94 (0.67, 1.33) | 1.06 (0.76, 1.47) | 0.84 (0.59, 1.21) | 0.5283 | 0.94 (0.81, 1.09) |  |

Model 1 was adjusted age, sex, ethnicity, educational attainment, and socioeconomic deprivation, body mass index, smoking status, alcohol drinking, healthy diet, leisure time physical activity, eGFR, family history of CVD, diabetes, hypertension, and hypercholesterolemia.

**Supplementary Table S9. Subgroup analysis of associations between CALLY index and** all-cause mortality i**n CKD patients**

|  |  | Quartiles of CALLY index | | | |  | Per 1-SD increase in CALLY index | P for interaction |
| --- | --- | --- | --- | --- | --- | --- | --- | --- |
|  | Subgroup | Q1 | Q2 | Q3 | Q4 | P for trend |  |  |
| Age | < 60 years | 1.00 (Ref.) | 0.77 (0.66, 0.91) | 0.59 (0.49, 0.70) | 0.46 (0.38, 0.56) | <0.001 | 0.88 (0.81, 0.96) | 0.077 |
|  | ≥ 60 years | 1.00 (Ref.) | 0.73 (0.67, 0.80) | 0.56 (0.51, 0.61) | 0.57 (0.51, 0.63) | <0.001 | 0.84 (0.79, 0.89) |  |
| Sex | Male | 1.00 (Ref.) | 0.70 (0.64, 0.78) | 0.54 (0.48, 0.61) | 0.53 (0.47, 0.60) | <0.001 | 0.84 (0.78, 0.89) | 0.321 |
|  | Female | 1.00 (Ref.) | 0.78 (0.69, 0.88) | 0.60 (0.53, 0.69) | 0.60 (0.52, 0.69) | <0.001 | 0.90 (0.84, 0.96) |  |
| Qualifications | No relevant qualifications | 1.00 (Ref.) | 0.75 (0.63, 0.90) | 0.52 (0.43, 0.63) | 0.52 (0.43, 0.64) | <0.001 | 0.87 (0.79, 0.95) | 0.603 |
|  | College or University degree | 1.00 (Ref.) | 0.68 (0.50, 0.92) | 0.50 (0.36, 0.69) | 0.42 (0.29, 0.61) | <0.001 | 0.83 (0.68, 1.00) |  |
|  | Others | 1.00 (Ref.) | 0.73 (0.67, 0.80) | 0.58 (0.53, 0.64) | 0.58 (0.52, 0.65) | <0.001 | 0.87 (0.82, 0.92) |  |
| Townsend deprivation index | Low | 1.00 (Ref.) | 0.78 (0.69, 0.87) | 0.58 (0.51, 0.66) | 0.54 (0.47, 0.62) | <0.001 | 0.89 (0.83, 0.95) | 0.458 |
|  | High | 1.00 (Ref.) | 0.71 (0.64, 0.78) | 0.56 (0.50, 0.63) | 0.57 (0.50, 0.64) | <0.001 | 0.84 (0.79, 0.90) |  |
| Body mass index | < 30 kg/m^2^ | 1.00 (Ref.) | 0.72 (0.64, 0.80) | 0.56 (0.50, 0.63) | 0.56 (0.50, 0.62) | <0.001 | 0.91 (0.86, 0.96) | 0.611 |
|  | ≥ 30 kg/m^2^ | 1.00 (Ref.) | 0.77 (0.69, 0.86) | 0.58 (0.51, 0.67) | 0.63 (0.53, 0.75) | <0.001 | 0.80 (0.72, 0.89) |  |
| Smoking status | Never | 1.00 (Ref.) | 0.73 (0.64, 0.83) | 0.53 (0.46, 0.61) | 0.54 (0.46, 0.62) | <0.001 | 0.91 (0.85, 0.97) | 0.761 |
|  | Past or current | 1.00 (Ref.) | 0.75 (0.68, 0.83) | 0.59 (0.53, 0.66) | 0.57 (0.50, 0.64) | <0.001 | 0.83 (0.77, 0.88) |  |
| Alcohol | Never | 1.00 (Ref.) | 0.77 (0.56, 1.06) | 0.44 (0.30, 0.64) | 0.69 (0.48, 0.99) | 0.0021 | 0.89 (0.73, 1.07) | 0.158 |
|  | Past or current | 1.00 (Ref.) | 0.73 (0.67, 0.79) | 0.57 (0.52, 0.62) | 0.54 (0.49, 0.60) | <0.001 | 0.86 (0.82, 0.90) |  |
| Leisure time physical activity | < 500 MET mins/week | 1.00 (Ref.) | 0.75 (0.67, 0.85) | 0.54 (0.47, 0.62) | 0.56 (0.48, 0.65) | <0.001 | 0.91 (0.85, 0.98) | 0.676 |
|  | ≥ 500 MET mins/week | 1.00 (Ref.) | 0.76 (0.68, 0.86) | 0.59 (0.52, 0.67) | 0.54 (0.47, 0.62) | <0.001 | 0.84 (0.78, 0.90) |  |
| Healthy diet | No | 1.00 (Ref.) | 0.73 (0.66, 0.79) | 0.57 (0.52, 0.63) | 0.54 (0.49, 0.61) | <0.001 | 0.86 (0.82, 0.91) | 0.145 |
|  | Yes | 1.00 (Ref.) | 0.84 (0.70, 1.01) | 0.51 (0.41, 0.63) | 0.58 (0.47, 0.72) | <0.001 | 0.81 (0.73, 0.91) |  |
| eGFR | Low | 1.00 (Ref.) | 0.70 (0.63, 0.78) | 0.52 (0.46, 0.58) | 0.47 (0.41, 0.53) | <0.001 | 0.77 (0.72, 0.83) | 0.002 |
|  | High | 1.00 (Ref.) | 0.78 (0.69, 0.88) | 0.63 (0.55, 0.72) | 0.67 (0.59, 0.77) | <0.001 | 0.94 (0.89, 1.00) |  |
| Family history of CVD | No | 1.00 (Ref.) | 0.76 (0.69, 0.83) | 0.57 (0.51, 0.64) | 0.60 (0.53, 0.67) | <0.001 | 0.89 (0.84, 0.94) | 0.222 |
|  | Yes | 1.00 (Ref.) | 0.71 (0.62, 0.80) | 0.55 (0.48, 0.64) | 0.49 (0.42, 0.57) | <0.001 | 0.82 (0.76, 0.89) |  |
| Diabetes | No | 1.00 (Ref.) | 0.74 (0.67, 0.80) | 0.58 (0.53, 0.64) | 0.56 (0.51, 0.62) | <0.001 | 0.86 (0.81, 0.91) | 0.653 |
|  | Yes | 1.00 (Ref.) | 0.74 (0.63, 0.88) | 0.51 (0.43, 0.62) | 0.53 (0.44, 0.65) | <0.001 | 0.89 (0.81, 0.98) |  |
| Hypertension | No | 1.00 (Ref.) | 0.77 (0.69, 0.87) | 0.59 (0.52, 0.68) | 0.56 (0.49, 0.65) | <0.001 | 0.89 (0.84, 0.95) | 0.732 |
|  | Yes | 1.00 (Ref.) | 0.71 (0.65, 0.79) | 0.55 (0.49, 0.62) | 0.56 (0.49, 0.63) | <0.001 | 0.84 (0.78, 0.90) |  |
| Hyperlipidemia | No | 1.00 (Ref.) | 0.75 (0.68, 0.82) | 0.57 (0.51, 0.63) | 0.54 (0.49, 0.60) | <0.001 | 0.85 (0.80, 0.90) | 0.697 |
|  | Yes | 1.00 (Ref.) | 0.70 (0.59, 0.82) | 0.55 (0.47, 0.66) | 0.58 (0.48, 0.69) | <0.001 | 0.89 (0.81, 0.97) |  |

Model 1 was adjusted age, sex, ethnicity, educational attainment, and socioeconomic deprivation, body mass index, smoking status, alcohol drinking, healthy diet, leisure time physical activity, eGFR, family history of CVD, diabetes, hypertension, and hypercholesterolemia.

**Supplementary Table S10. Subgroup analysis of associations between CALLY index and** CVD-specific mortality i**n CKD patients**

|  |  | Quartiles of CALLY index | | | |  | Per 1-SD increase in CALLY index | P for interaction |
| --- | --- | --- | --- | --- | --- | --- | --- | --- |
|  | Subgroup | Q1 | Q2 | Q3 | Q4 | P for trend |  |  |
| Age | < 60 years | 1.00 (Ref.) | 0.75 (0.53, 1.05) | 0.48 (0.32, 0.73) | 0.45 (0.29, 0.68) | <0.001 | 0.79 (0.63, 0.99) | 0.859 |
|  | ≥ 60 years | 1.00 (Ref.) | 0.81 (0.68, 0.97) | 0.52 (0.42, 0.64) | 0.55 (0.44, 0.69) | <0.001 | 0.82 (0.73, 0.93) |  |
| Sex | Male | 1.00 (Ref.) | 0.77 (0.63, 0.93) | 0.51 (0.41, 0.64) | 0.51 (0.40, 0.65) | <0.001 | 0.78 (0.68, 0.90) | 0.673 |
|  | Female | 1.00 (Ref.) | 0.87 (0.66, 1.13) | 0.53 (0.39, 0.73) | 0.63 (0.45, 0.87) | <0.001 | 0.91 (0.77, 1.07) |  |
| Qualifications | No relevant qualifications | 1.00 (Ref.) | 0.81 (0.57, 1.14) | 0.46 (0.31, 0.69) | 0.49 (0.33, 0.73) | <0.001 | 0.82 (0.67, 1.01) | 0.960 |
|  | College or University degree | 1.00 (Ref.) | 0.95 (0.48, 1.89) | 0.48 (0.21, 1.08) | 0.58 (0.23, 1.43) | 0.0708 | 1.10 (0.87, 1.38) |  |
|  | Others | 1.00 (Ref.) | 0.76 (0.63, 0.92) | 0.53 (0.42, 0.66) | 0.55 (0.43, 0.70) | <0.001 | 0.81 (0.71, 0.93) |  |
| Townsend deprivation index | Low | 1.00 (Ref.) | 0.99 (0.78, 1.27) | 0.55 (0.41, 0.73) | 0.58 (0.43, 0.78) | <0.001 | 0.89 (0.77, 1.03) | 0.121 |
|  | High | 1.00 (Ref.) | 0.68 (0.55, 0.84) | 0.51 (0.40, 0.65) | 0.53 (0.40, 0.69) | <0.001 | 0.77 (0.65, 0.90) |  |
| Body mass index | < 30 kg/m^2^ | 1.00 (Ref.) | 0.78 (0.62, 0.98) | 0.45 (0.35, 0.58) | 0.50 (0.39, 0.64) | <0.001 | 0.82 (0.72, 0.94) | 0.295 |
|  | ≥ 30 kg/m^2^ | 1.00 (Ref.) | 0.83 (0.67, 1.04) | 0.62 (0.47, 0.82) | 0.65 (0.47, 0.91) | <0.001 | 0.87 (0.72, 1.05) |  |
| Smoking status | Never | 1.00 (Ref.) | 0.67 (0.51, 0.87) | 0.46 (0.34, 0.62) | 0.48 (0.36, 0.66) | <0.001 | 0.84 (0.72, 0.99) | 0.440 |
|  | Past or current | 1.00 (Ref.) | 0.88 (0.72, 1.07) | 0.55 (0.43, 0.69) | 0.56 (0.43, 0.73) | <0.001 | 0.80 (0.69, 0.93) |  |
| Alcohol | Never | 1.00 (Ref.) | 0.70 (0.40, 1.24) | 0.35 (0.17, 0.74) | 0.50 (0.24, 1.02) | 0.0077 | 0.91 (0.63, 1.32) | 0.885 |
|  | Past or current | 1.00 (Ref.) | 0.80 (0.68, 0.94) | 0.53 (0.44, 0.64) | 0.54 (0.44, 0.66) | <0.001 | 0.82 (0.73, 0.91) |  |
| Leisure time physical activity | < 500 MET mins/week | 1.00 (Ref.) | 0.76 (0.59, 0.97) | 0.39 (0.29, 0.54) | 0.47 (0.34, 0.66) | <0.001 | 0.78 (0.64, 0.94) | 0.309 |
|  | ≥ 500 MET mins/week | 1.00 (Ref.) | 0.85 (0.67, 1.09) | 0.59 (0.45, 0.77) | 0.54 (0.40, 0.72) | <0.001 | 0.87 (0.75, 1.00) |  |
| Healthy diet | No | 1.00 (Ref.) | 0.80 (0.67, 0.96) | 0.50 (0.40, 0.61) | 0.54 (0.43, 0.67) | <0.001 | 0.83 (0.73, 0.94) | 0.949 |
|  | Yes | 1.00 (Ref.) | 0.79 (0.54, 1.14) | 0.45 (0.29, 0.70) | 0.46 (0.29, 0.72) | <0.001 | 0.72 (0.55, 0.94) |  |
| eGFR | Low | 1.00 (Ref.) | 0.76 (0.62, 0.93) | 0.46 (0.36, 0.59) | 0.47 (0.36, 0.62) | <0.001 | 0.80 (0.69, 0.93) | 0.590 |
|  | High | 1.00 (Ref.) | 0.81 (0.63, 1.04) | 0.58 (0.44, 0.77) | 0.60 (0.45, 0.81) | <0.001 | 0.81 (0.69, 0.96) |  |
| Family history of CVD | No | 1.00 (Ref.) | 0.80 (0.65, 0.98) | 0.56 (0.44, 0.71) | 0.55 (0.43, 0.72) | <0.001 | 0.88 (0.77, 1.00) | 0.813 |
|  | Yes | 1.00 (Ref.) | 0.78 (0.61, 1.00) | 0.46 (0.35, 0.62) | 0.52 (0.39, 0.71) | <0.001 | 0.75 (0.63, 0.90) |  |
| Diabetes | No | 1.00 (Ref.) | 0.75 (0.63, 0.90) | 0.47 (0.38, 0.59) | 0.52 (0.41, 0.66) | <0.001 | 0.85 (0.75, 0.97) | 0.481 |
|  | Yes | 1.00 (Ref.) | 0.92 (0.67, 1.26) | 0.64 (0.45, 0.91) | 0.57 (0.39, 0.82) | <0.001 | 0.77 (0.63, 0.95) |  |
| Hypertension | No | 1.00 (Ref.) | 0.72 (0.55, 0.95) | 0.42 (0.30, 0.59) | 0.55 (0.40, 0.75) | <0.001 | 0.96 (0.83, 1.11) | 0.394 |
|  | Yes | 1.00 (Ref.) | 0.85 (0.70, 1.02) | 0.57 (0.46, 0.71) | 0.54 (0.42, 0.70) | <0.001 | 0.74 (0.64, 0.87) |  |
| Hyperlipidemia | No | 1.00 (Ref.) | 0.85 (0.71, 1.02) | 0.47 (0.37, 0.59) | 0.53 (0.42, 0.67) | <0.001 | 0.80 (0.70, 0.92) | 0.187 |
|  | Yes | 1.00 (Ref.) | 0.69 (0.50, 0.94) | 0.61 (0.44, 0.84) | 0.54 (0.38, 0.77) | <0.001 | 0.84 (0.69, 1.01) |  |

Model 1 was adjusted age, sex, ethnicity, educational attainment, and socioeconomic deprivation, body mass index, smoking status, alcohol drinking, healthy diet, leisure time physical activity, eGFR, family history of CVD, diabetes, hypertension, and hypercholesterolemia.


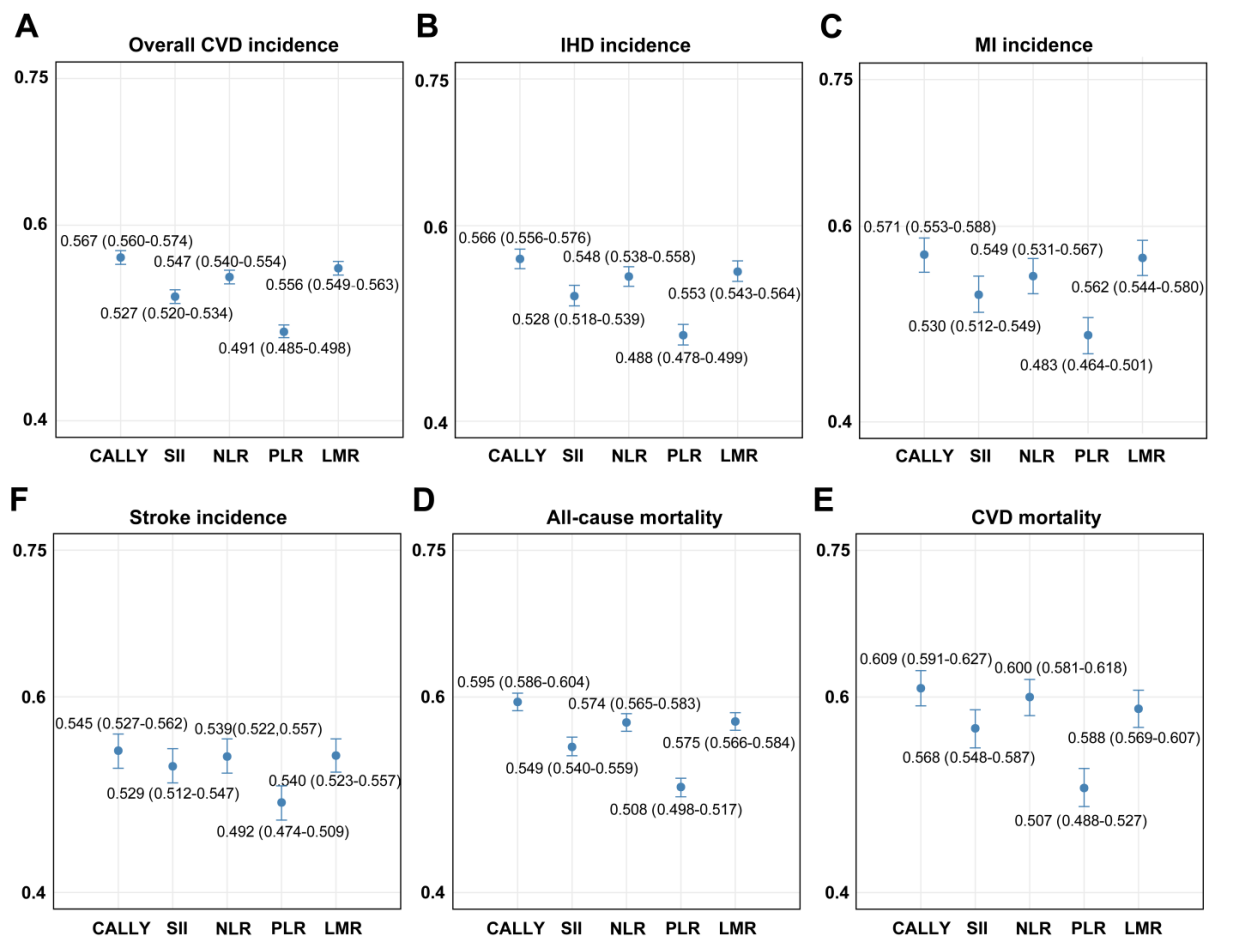


**Supplementary Figure S1.** The independent predictive capacity of CALLY and other inflammatory biomarkers for CVD incidence and all-cause and CVD-specific mortality i**n CKD patients**
